# PASSIFOR: A reference library of DNA barcodes for French saproxylic beetles (Insecta, Coleoptera)

**DOI:** 10.3897/BDJ.3.e4078

**Published:** 2015-03-04

**Authors:** Rodolphe Rougerie, Carlos Lopez-Vaamonde, Thomas Barnouin, Julien Delnatte, Nicolas Moulin, Thierry Noblecourt, Benoît Nusillard, Guillem Parmain, Fabien Soldati, Christophe Bouget

**Affiliations:** ‡ Muséum national d'Histoire Naturelle, Sorbonne Universités, Institut de Systématique, Évolution, Biodiversité (ISYEB), UMR 7205 – CNRS, MNHN, UPMC, EPHE, Paris, France; § INRA UR633 Zoologie Forestière, Orléans, France; | Office National des Forêts (ONF), Laboratoire National d'Entomologie Forestière, Quillan, France; ¶ Independent researcher, Avignon, France; # Entreprise Nicolas Moulin Entomologiste, Bihorel, France; ¤ IRSTEA Ecosystèmes Forestiers, Nogent-Sur-Vernisson, France

**Keywords:** DNA barcoding, COI, molecular identification, cryptic diversity, Coleoptera, forest insects, ecological indicators

## Abstract

Saproxylic beetles – associated with dead wood or with other insects, fungi and microorganisms that decompose it – play a major role in forest nutrient cycling. They are important ecosystem service providers and are used as key bio-indicators of old-growth forests. In France alone, where the present study took place, there are about 2500 species distributed within 71 families. This high diversity represents a major challenge for specimen sorting and identification.

The PASSIFOR project aims at developing a DNA metabarcoding approach to facilitate and enhance the monitoring of saproxylic beetles as indicators in ecological studies. As a first step toward that goal we assembled a library of DNA barcodes using the standard genetic marker for animals, i.e. a portion of the COI mitochondrial gene. In the present contribution, we release a library including 656 records representing 410 species in 40 different families. Species were identified by expert taxonomists, and each record is linked to a voucher specimen to enable future morphological examination. We also highlight and briefly discuss cases of low interspecific divergences, as well as cases of high intraspecific divergences that might represent cases of overlooked or cryptic diversity.

## Introduction

Forests ecosystems cover nearly 30% of the total land surface globally and host most of the terrestrial biodiversity. They are highly complex systems whose functioning and sustainability depends on a range of spatially and temporally dynamic abiotic and biotic factors. To monitor or diagnose forest ecosystems, ecologists have historically used both physico-chemical and biological indicators. Among the latters, saproxylic beetles – associated with dead wood or with other insects, fungi and microorganisms that decompose it – have been used as key bio-indicators of old-growth forests in both temperate and boreal regions of the globe (but see Grove and Stork (1999) for perspectives toward their monitoring in tropical forests). Their diversity is high (several hundred species co-occur in most forests), they can be abundant, and samples are generally easily collected using standard techniques facilitating comparisons between sites. Saproxylic beetle species include both generalists and highly specialized organisms, sometimes requiring complex and stringent conditions in order to fulfill their development and reproduction. As a consequence, their communities have been shown to be tightly linked to the features and the dynamics of the habitat (Grove 2002).

In conservation biology studies, saproxylic beetles have often been studied through the perspective of focal species (often endangered/patrimonial species (e.g. Buse et al. 2007)), and of communities (e.g. Bouget et al. 2014, Buse et al. 2010, Lassauce et al. 2013, Quinto et al. 2012), considering the presence/absence of the former, and/or the diversity and relative abundances of species within the latter. However, these studies are strongly impeded by the considerable diversity of these insects. In France alone, there are about 2500 species distributed within 71 families, and several hundreds of specimens representing dozens of species can be collected in a single trap (see Bouget et al. (2009) for details about standard collecting methods using interception traps). Because of this diversity and abundance, and because species identification using morphology requires strong and rather scarce taxonomic expertise, specimen sorting and identification represent the main bottleneck in studies of saproxylic beetles, thus impeding their consideration in large-scale forest biodiversity monitoring schemes.

DNA-based identification, and the development of metagenomic approaches using Next Generation Sequencing (NGS) technologies, hold strong promise to overcome this impediment and may alleviate funding and time constraints for large-scale studies on these insects. Molecular identification of species has seen a considerable and rapid development over the past decade following the introduction of DNA barcoding by Hebert et al. (2003); during this period, the field has experienced extensive testing in a large variety of organisms, including many insect orders. DNA barcode libraries are being developed at a steady pace, combining genetic data (usually the sequences of the genetic marker used as the standard DNA barcode in animals: a 658bp fragment of the mtDNA COI gene, although additional markers are sometimes used to complement it), taxonomic information, and specimen data (collecting information, voucher repository, images). A global online database, the Barcode of Life Datasystems (BOLD), serves as the central repository for these libraries (www.boldsystems.org) and combines classical database features with a workbench facilitating data analyses and data sharing (Ratnasingham and Hebert 2007). At the same time, advances in NGS technologies have increased by several orders of magnitude the yield and throughput of DNA sequencing and triggered the development of metagenomics. Multiple genomes can now be extracted, amplified and sequenced simultaneously, allowing for the sequencing of environmental (air, water or soil for instance) or bulk (complex assemblages of multiple individuals) samples (Shokralla et al. 2012, Tautz et al. 2010). By targeting a DNA marker that permits species identification, like DNA barcodes, this method can be used to document the species composition of complex samples, like communities, in an approach called DNA metabarcoding (Hajibabaei et al. 2011, Taberlet et al. 2012, Yu et al. 2012, Cristescu 2014).

The PASSIFOR project, initiated by the National Research Institute of Science and Technology for Environment and Agriculture (IRSTEA) and by the National Institute for Agricultural Research (INRA), aims at developing a DNA metabarcoding approach for French species of saproxylic beetles to facilitate and enhance the use of these insects as forest indicators. As a first step toward that goal, we present and release in this paper a DNA barcode reference library for these insects, including 656 records representing 410 species in 251 genera and 40 different families. This library represents about 16% of the national fauna and we expect that its development in the next future will further contribute to the assembly of a DNA barcode library for European beetles. Remarkable progress was recently accomplished toward that goal with the published results of national campaigns in Finland (Pentinsaari et al. 2014) and in Germany (Hendrich et al. 2014) together representing 4330 species with DNA barcodes in Northern and Central Europe.

## General description

### Purpose

This library aims to provide an authoritative reference library for the DNA-based species identification of French saproxylic beetles, in order to facilitate the use of DNA metabarcoding in biodiversity monitoring networks focusing on these forest insects. It is also expected to develop the use of DNA barcodes by the community of coleopterists, in combination with characters from the morphology, ecology and biogeography of species, to address taxonomic questions.

### Additional information

Because the available funding for this project was too limited to develop an exhaustive library for French saproxylic beetles (ca. 2500 species) or to allow the documentation of intraspecific and geographical patterns of genetic variation, our initial objective has been to target a broad taxonomic coverage, favoring taxonomic diversity at the family, genus and species levels. Only a few species complex or notoriously difficult genera (e.g. *Ampedus* in family Elateridae) were more densely sampled.

The PASSIFOR library uses the standard DNA barcode for animals, i.e. a 658bp fragment of the COI mitochondrial gene.

Species identifications were provided by expert taxonomists for these groups. All records were initially identified on the basis of morphological examination, and voucher specimens are preserved in the collections of the taxonomists as references for these records. Any future change in the taxonomy/nomenclature of these insects will be reported in the PASSIFOR library, after authoritative validation by the taxonomists.

## Project description

### Title

PASSIFOR: stands for (in French) "Proposition d'Amélioration du Système de Suivi de la bIodiversité FORestière": Proposal toward improving monitoring of forest biodiversity.

### Personnel

*PIs*: Christophe Bouget (IRSTEA, Nogent-Sur-Vernisson) & Carlos Lopez-Vaamonde (INRA, Orléans)

*Postdoctoral fellow*: Rodolphe Rougerie (INRA, Orléans)

### Study area description

Western Europe: France (99.1% of the samples), Czech Republic, Italy, Spain, and Morocco.

### Funding

This project is supported by a grant from the French Ministry of Agriculture (MAAF) to IRSTEA (CB) and INRA (CLV). Sequencing of DNA barcodes also benefitted from funding by Genome Canada and the Ontario Genomics Institute (OGI) to the International Barcode of Life Project (iBOL).

## Sampling methods

### Study extent

The PASSIFOR library focuses on French species within 40 different families of saproxylic beetles.

### Sampling description

Tissue samples for DNA extraction were collected mostly from dry collection specimens; only a limited number of samples were preserved in 95%-ethanol. All specimens were photographed and specimen data were compiled in excel spreadsheets for submission to BOLD.

Most specimens were sampled by RR and CLV in the National Laboratory for Forest Entomology, Quillan, France. GP, TB and BN assisted in sampling and in databasing records in their institutional collections. NM sampled specimens in his own reference collection, while JD selected and shipped a selection of specimens (Elateridae, especially members of genus *Ampedus*) to INRA Orleans where RR handled tissue sampling, photography and databasing.

### Quality control

All tissue samples were assembled in 96-well plates in which one well (location H12) was left empty to serve as a negative control. After sequencing and upload of sequences into BOLD, DNA barcodes were compared through classical analyses of genetic distances (blast hits, NJ trees) to conspecific records, when existing, in other accessible DNA barcoding projects/campaigns. Discordances between DNA results and taxonomy derived from morphology (DNA barcodes shared by distinct species, deep intra-specific splits (>2%)) led to re-examination of the specimens; collegial discussions were initiated to address these issues by revealing possible cases of mis-identification or cross-contamination.

### Step description

The construction of the PASSIFOR library can be divided into two main steps:

1. *Specimen sampling and data compilation*:
  - tissue sampling. Using flame-decontaminated forceps, we usually pulled one leg from each specimen sampled. Occasionally, when these appendages were difficult to reach, we used the antenna or hindwing of the insect. For the smallest species, we sometimes used up to three of these body parts (usually several legs). Only in one case, a tiny representative of the Scolytinae *Ernoporicus fagi *, did we use the whole insect and as a consequence did not preserve any voucher specimen.
  - photography. Each specimen was photographed individually along with a scale.
  - data compilation. We used standard BOLD spreadsheets to compile:
    - voucher information: SampleID (a unique BOLD identifier for the specimen; also added on a label pinned with the voucher specimen) and institution storing.
    - Taxonomy data: higher level taxonomy; species identification; identifier, including contact information.
    - Specimen details: sex (when available); reproduction mode; life stage; type of tissue used (for most specimens); collecting method (when available).
    - Collection data: collectors; date collected; country; administrative region and department; sector; exact site; latitude, longitude and elevation (when available).
  - upload to BOLD. Following the standard BOLD procedure for DNA barcode library construction, we created a dedicated project in BOLD. This project (code PSFOR, publicly accessible) hosts records for all the samples processed (including failures), whereas the actual PASSIFOR library (dataset DS-PSFOR01, see the *Data resources* section below) only includes records successfully sequenced and subsequently validated by taxonomists.
2. *Sequencing of DNA barcodes*: The Canadian Centre for DNA Barcoding (CCDB), hosted by the Biodiversity Institute of Ontario (BIO) at the University of Guelph, Ontario, Canada) processed the tissue samples; all operations were carried out following the standard high-throughput protocols in place at CCDB and available from http://ccdb.ca/resources.php. For PCR amplification, we used a primer cocktail combining the LCO1490/HCO2198 pair (Folmer et al. 1994) with the LepF1/LepR1 pair (Hebert et al. 2004) for amplification of the full-length (658bp) DNA barcode region of the COI gene. Samples failing to amplify with these primers were alternatively processed using internal primers targeting shorter fragment; MLepR2 (Hebert et al. 2013) was used along with LCO1490/LepF1, and MLepF1 (Hajibabaei et al. 2006) was used with HCO1498/LepR1 to target fragments of 307bp and 407bp, respectively. Unpurified PCR fragments were sequenced in both directions using an ABI 3730XL DNA Analyzer (Applied Biosystems, Foster City, CA, USA). CodonCode (CodonCode Corporation, Centerville, MA) was used for trimming primers, contig assembly and sequence editing; alignment was straightforward in absence of indels and the sequences, along with corresponding trace files, were uploaded to BOLD.

## Geographic coverage

### Description

**Figure 1.**
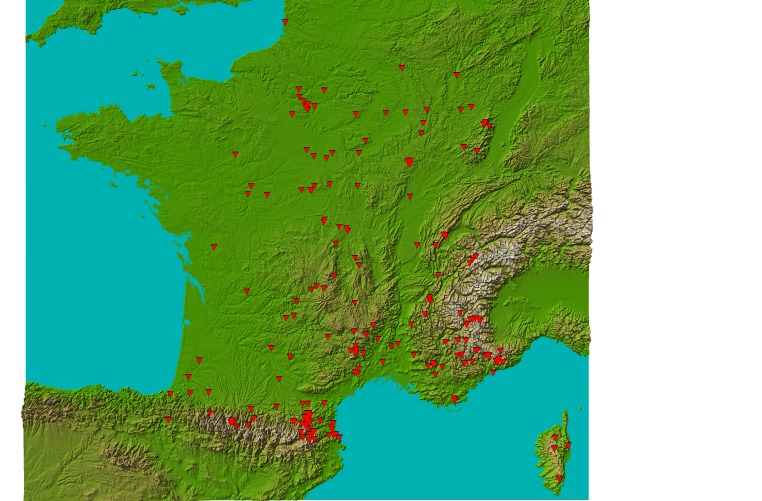
Distribution of the PASSIFOR library records (Suppl. material 1).

The PASSIFOR library covers 17 of the 22 administrative regions of France, including Corsica. The map in Fig. 1 represents the distribution of the PASSIFOR records.

### Coordinates

41.7 and 50.5 Latitude; -1.6 and 9.5 Longitude.

## Taxonomic coverage

### Description

The PASSIFOR library comprises 656 records for saproxylic beetles belonging to 40 different families. They represent 410 species in 251 genera. Table 1 provides the details on sampling for each family.

The nomenclature used generally follows that in the eight volumes of the Catalogue of Palaearctic Coleoptera series, edited by Löbl and Smetana (see f.i Löbl and Smetana (2003)), which in turn was largely followed, for French beetles, in the recent national catalogue by Tronquet (2014). New names and nomenclatural changes after publication of the volumes of the Löbl & Smetana catalogue were sometimes adopted in the PASSIFOR library, but only if they did not conflict with other DNA barcode libraries for these insects, or if they are considered consensual within the community of coleopterists involved in the construction of these libraries. This strategy favors the consistency of names used within several independently constructed libraries in BOLD rather than an authoritative stand for one or another of alternative names. This should prevent, or at least limit, the existence of "parallel taxonomies" (multiple names or combination of names for a single species) in BOLD.

## Usage rights

### Use license

Open Data Commons Attribution License

## Data resources

### Data package title

PASSIFOR DNA barcode reference library

### Resource link

http://dx.doi.org/10.5883/DS-PSFOR01

### Alternative identifiers

PASSIFOR library

### Number of data sets

1

### Data set 1.

#### Data set name

DS-PSFOR01

#### Data format

xml, tsv, fasta, ab1

#### Number of columns

45

#### Download URL

http://www.boldsystems.org/index.php/Public_BINSearch?searchtype=records

#### Description

The PASSIFOR library dataset can be downloaded from the Public Data Portal of BOLD in different formats (data as xml or tsv files, sequences and trace files as fasta and ab1 files). Alternatively, BOLD users can login and access the dataset via the Workbench platform of BOLD (see the public dataset list in the User Console page, under the name of first author); all records are also searchable within BOLD using the search function of the database.

The version of the library at the time of writing of this manuscript is also included as Suppl. materials 1, 2 in the form of an excel spreadsheet for record information and of a fasta file containing all aligned sequences.

**Data set 1.** 

Column label	Column description
processid	Unique identifier for the DNA sample.
sampleid	Unique identifier for the specimen and by extension the tissue sample used for DNA analysis.
recordID	Entry number in the database.
catalognum	Identifier for specimen assigned by formal collection upon accessioning.
fieldnum	Identifier for specimen assigned in the field.
institution_storing	The full name of the institution that has physical possession of the voucher specimen.
bin_uri	URI (Unique Resource Identifier) for the Barcode Index Number (BIN) to which the record belongs.
phylum_taxID	Taxonomic identifier of level Phylum
phylum_name	Phylum name
class_taxID	Taxonomic identifier of level Class
class_name	Class name
order_taxID	Taxonomic identifier of level Order
order_name	Order name
family_taxID	Taxonomic identifier of level Family
family_name	Family name
subfamily_taxID	Taxonomic identifier of level Subfamily
subfamily_name	Subfamily name
genus_taxID	Taxonomic identifier of level Genus
genus_name	Genus name
species_taxID	Taxonomic identifier of level Species
species_name	Species name
identification_provided_by	Full name of primary individual who assigned the specimen to a taxonomic group.
voucher_type	Status of the specimen in an accessioning process.
tissue_type	A brief description of the type of tissue or material analyzed.
collectors	The full or abbreviated names of the individuals or team responsible for collecting the sample in the field.
collectiondate	The date during which the sample was collected.
collectiondate_accuracy	A numerical representation of the precision of the Collection Date given in days and is represented as +/- the value.
lifestage	The age class or life stage of the specimen at the time of sampling.
sex	The sex of the specimen.
reproduction	The presumed method of reproduction.
extrainfo	A brief note or project term associated with the specimen for rapid analysis.
notes	General notes regarding the specimen.
lat	The geographic latitude (in decimal degrees) of the geographic center of a location.
lon	The geographic longitude (in decimal degrees) of the geographic center of a location.
coord_source	The source of the latitude and longitude measurements.
coord_accuracy	A decimal representation of the precision of the coordinates given in the decimalLatitude and decimalLongitude.
elev	Elevation of sampling site. Measured in meters relative to sea level. Negative values indicate a position below sea level.
depth	For organisms collected beneath the surface of a water body. Measured in meters below surface of water.
elev_accuracy	A numerical representation of the precision of the elevation given in meters and is represented as +/- the elevation value.
depth_accuracy	A numerical representation of the precision of the depth given in meters and is represented as +/- the depth value.
country	The full, unabbreviated name of the country, major political unit, or ocean in which the organism was collected.
province	The full, unabbreviated name of the state, province, territory, or prefecture (i.e., the next smallest political region below Country) in which the organism was collected.
region	The full, unabbreviated name of the county, shire, municipality, or park (i.e., the next smallest political region below province/state) in which the organism was collected.
sector	The full, unabbreviated name of the lake, conservation area or sector of park in which the organism was collected.
exactsite	Additional text descriptions regarding the exact location of the collection site relative to a geographic or biologically relevant landmark.

## Additional information

In the following sections we provide a quick description of the results of DNA barcode analyses as carried out using the analytical tools available through the BOLD's workbench at the time of writing of this manuscript.

### Sequence composition

The summary statistics for nucleotide frequency distribution are provided in Table 2. The range of variation in GC content (26 - 47%) within our very diverse set of taxa (40 families) is large and similar to previous reports in insects (Clare et al. 2008​). It is most variable at the 1^st ^(34.6 - 54.7%) and 3^rd^ (1.9 - 43.8%) codon positions.

### Analyses of genetic distances

All sequence analyses were carried out in BOLD using Kimura-2 parameters (K2P) distances with BOLD handling the sequence alignment. Alternative alignment methods were tested (including the use of sequences aligned "as uploaded") and proved to have no impact on the results.

**Figure 2.**
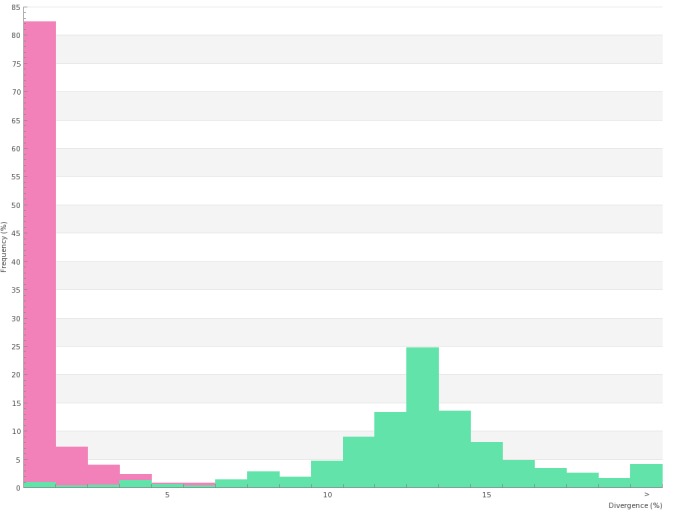
Frequency distribution of within-species (normalized, in pink) and within-genus (green) K2P distances for records of the PASSIFOR library (sequences longer than 400 bp only: 597 records, 388 species). Table of distances is provided as Suppl. material 4 and Suppl. material 5.

**Figure 3.**
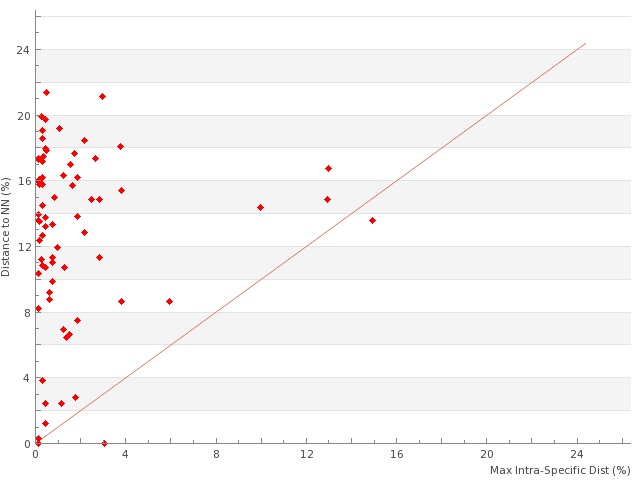
Scatterplot representing for each species of the PASSIFOR library (sequences longer than 400 bp only: 125 species after exclusion of singletons) the minimum distance to Nearest Neighbour (NN) plotted against the maximum intra-specific distance (Suppl. material 6).

All 656 sequences of the library where used to build a Neighbor-Joining (NJ) tree as illustrated in Suppl. material 3. For the analysis of intraspecific and interspecific distances, we reduced the dataset to sequences longer than 400bp (597 records, 388 species). General summary statistics at the species, genus and family levels are given in Table 3; Fig. 2 shows the frequency distributions of genetic distances within species (normalized) and within genus. Fig. 3 represents the distribution of maximum intraspecific distances (singletons excluded) plotted against distances to Nearest Neighbour within the library. Overall, we observe a conspicuous bimodal pattern suggesting the existence of a marked "barcode gap" between intraspecific and interspecific genetic divergence. We note however that in the vast majority of species our sampling remains too limited, both taxonomically (sister species often unsampled) and numerically (intraspecific divergence undocumented for most species) to test the extent of this gap and its consistency.

### Discrepancies between current taxonomy and DNA barcode results

While we are aware of the limitation of our dataset to address taxonomic questions in cases where DNA barcodes and current taxonomy reveal a possible discordance, we report here two categories of apparent conflicts between the results from DNA barcode analyses and species identifications derived from morphology.

1. High intraspecific divergence (>2%) were observed in 7 species (Table 4). All these cases require further sampling and investigation to figure if they represent cases of overlooked or cryptic diversity, or if they may represent geographical population structure, ancestral polymorphisms, or variation resulting from *Wolbachia* infections (Smith et al. 2012). As an example, in the Tenebrionidae *Nalassus ecoffeti*, where intraspecific genetic distance is as high as 13.2%, our results suggest the possible validity of the currently synonymized Pyrenean species *N. temperei* Ardoin, 1958 (F. Soldati, personal communication).
2. Low interspecific divergences (<2%) were observed in 6 pairs of species, 1 triplet, and 2 pairs of subspecies (Table 5). In total, of the 410 species sampled in the PASSIFOR library, 15 (3.6%) fall in this category of low to null interspecific distances. Here again, these cases will require additional sampling and further investigation to understand if our results reflect cases of overlooked synonymy (as may be the case in the pairs *Ampedus pomorum* / *A. nemoralis*, *Anastrangalia dubia* / *A. reyi* (the second originally described as a mere variety of the former)), introgression through past or ongoing hybridization, or recent speciation resulting in low level of divergence (e.g. in the pairs *Pityophagus ferrugineus* / *P. laevior* and *Ampedus pomonae* / *A. sanguinolentus*). In fact, our results only revealed two cases of strictly shared DNA barcodes (one pair and one triplet within the taxonomically difficult genus *Ampedus*), although results for Central European samples of *Anastrangalia dubia* and *A. reyi* (Hendrich et al. 2014) confirmed that the two species cannot be distinguished using their DNA barcodes.

## Supplementary Material

Supplementary material 1 PASSIFOR library - specimen and sequence data Data type: Record information - specimen data and sequence summary
Brief description: This excel spreadsheet includes information about all records in BOLD for the PASSIFOR library at the time of writing. It contains specimen data and sequence information, including GenBank accession numbers.
File: oo_30873.xls Rougerie R, Lopez-Vaamonde C, Barnouin T, Delnatte J, Moulin N, Noblecourt T, Nusillard B, Parmain G, Soldati F, Bouget C

Supplementary material 2 PASSIFOR library - DNA sequences Data type: Genomic data, DNA sequences
Brief description: Sequences in fasta format for the fragment of the COI mtDNA gene used as a standard DNA barcode in animals. Each sequence is identified by a chain of characters consisting of, in the following order and separated by pipes: processID, sampleID, species_name, DNA marker
File: oo_30874.fasta Rougerie R, Lopez-Vaamonde C, Barnouin T, Delnatte J, Moulin N, Noblecourt T, Nusillard B, Parmain G, Soldati F, Bouget C

Supplementary material 3 Neighbour Joining tree reconstructed from the 656 DNA barcodes of the PASSIFOR library. Data type: Distance tree
Brief description: NJ tree resulting from the analysis with BOLD of the 656 DNA barcode sequences of the PASSIFOR library. Parameters for tree reconstruction are as follow: distance model: K2P; alignment method: BOLD aligner; sequence length: >200 bp; pairwise deletion option; all three codon positions included.
File: oo_30875.pdf Rougerie R, Lopez-Vaamonde C, Barnouin T, Delnatte J, Moulin N, Noblecourt T, Nusillard B, Parmain G, Soldati F, Bouget C

Supplementary material 4 Pairwise K2P distances within species Data type: Genetic distances
Brief description: This table lists K2P distances for all pairwise comparisons between conspecific records in the PASSIFOR library (only DNA barcodes longer than 400bp); distances are calculated in BOLD (www.boldsystems.org).
File: oo_31675.xls Rougerie R, Lopez-Vaamonde C, Barnouin T, Delnatte J, Moulin N, Noblecourt T, Nusillard B, Parmain G, Soldati F, Bouget C

Supplementary material 5 Pairwise K2P distances within genera Data type: Genetic distances
Brief description: For the PASSIFOR library (only DNA barcodes longer than 400bp), this table lists K2P distances for all pairwise comparisons between heterospecific records of the same genus; distances are calculated in BOLD (www.boldsystems.org).
File: oo_31676.xls Rougerie R, Lopez-Vaamonde C, Barnouin T, Delnatte J, Moulin N, Noblecourt T, Nusillard B, Parmain G, Soldati F, Bouget C

Supplementary material 6 Intra-specific distances and distances to nearest neighbor (NN) Data type: Genetic distances
Brief description: This table provides, for each species of the PASSIFOR library with sequences longer than 400bp, mean and maximum intraspecific distances (non-applicable (N/A) for species represented as singletons in our dataset) as well as the distance to nearest neighbor (NN) within the library and its identification.
File: oo_39058.xlsx Rougerie R, Lopez-Vaamonde C, Barnouin T, Delnatte J, Moulin N, Noblecourt T, Nusillard B, Parmain G, Soldati F, Bouget C

## Figures and Tables

**Table 1.** Taxonomic coverage of the PASSIFOR library giving details of the number of records, genera and species sampled within each of the 40 families included (ordered alphabetically).

**Family**	**Records**	**Genera**	**Species**
Anthribidae	11	9	9
Biphyllidae	2	1	1
Bostrichidae	1	1	1
Brentidae	1	1	1
Buprestidae	7	6	7
Cerambycidae	165	69	115
Cerophytidae	1	1	1
Ciidae	1	1	1
Cleridae	12	5	6
Curculionidae	54	19	31
Elateridae	151	22	57
Endomychidae	2	1	1
Erotylidae	2	2	2
Eucinetidae	1	1	1
Eucnemidae	8	4	5
Histeridae	1	1	1
Laemophloeidae	2	2	2
Leiodidae	3	2	2
Lucanidae	8	4	4
Lycidae	6	5	5
Lymexylidae	4	2	2
Melandryidae	24	12	16
Monotomidae	9	1	7
Mycetophagidae	15	4	11
Nitidulidae	7	3	6
Nosodendridae	1	1	1
Oedemeridae	11	4	8
Prostomidae	1	1	1
Ptinidae	30	12	23
Pyrochroidae	5	2	3
Pythidae	1	1	1
Salpingidae	14	6	11
Scarabaeidae	16	5	14
Silvanidae	2	2	2
Sphindidae	2	2	2
Tenebrionidae	51	21	32
Tetratomidae	1	1	1
Trogidae	2	1	1
Trogossitidae	14	8	9
Zopheridae	7	5	6
**Total**	**656**	**251**	**410**

**Table 2.** Nucleotide frequency distribution for sequences (>400bp, 597 sequences analyzed) in the PASSIFOR library.

	Min	Mean	Max	SE
**G %**	13.37	16.17	21.73	0.04
**C %**	12.61	19.82	27.68	0.13
**A %**	25.31	29.74	34.15	0.06
**T %**	26.44	34.26	44.07	0.15
**GC %**	25.99	35.99	46.81	0.14
**GC % Codon Pos 1**	34.65	46.84	54.72	0.13
**GC % Codon Pos 2**	38.3	42.65	46.44	0.04
**GC % Codon Pos 3**	1.94	18.43	43.77	0.31

**Table 3.** Summary of distance (K2P) variations at species, genus and family levels, as calculated with BOLD from 597 records of the PASSIFOR library with DNA barcodes longer than 400bp.

	n	Taxa	Comparisons	Min Dist(%)	Mean Dist(%)	Max Dist(%)	SE Dist(%)
Within Species	334	125	458	0	0.85	14.93	0
Within Genus	362	73	3152	0	12.5	27.14	0
Within Family	573	25	20617	9.54	21.9	39.13	0

**Table 4.** List of species within the PASSIFOR library (sequence length>400 bp; 597 records, 388 species) with more than 2% intraspecific divergence (N = number of records).

**Family**	**Species**	**N**	**Max. Intrasp. (%)**
Cerambycidae	*Alosterna tabacicolor*	3	11.2
Cerambycidae	*Tetrops praeustus*	2	11.8
Cleridae	*Thanasimus formicarius*	2	11.5
Cleridae	*Tillus elongatus*	4	8.8
Elateridae	*Melanotus castanipes*	2	5.7
Elateridae	*Melanotus villosus*	3	4.5
Tenebrionidae	*Nalassus ecoffeti*	5	13.2

**Table 5.** List of species and subspecies pairs/triplet within the PASSIFOR library for which the minimum distance to the nearest heterospecific or heterosubspecific record is below 2% (number of records for each taxon is given within brackets next to its name).

**Family**	**Species pairs & triplet**	**Min. intersp. (%)**
Cerambycidae	*Anastrangalia dubia* (3) / *A. reyi* (1)	0.47
Cerambycidae	*Chlorophorus ruficornis* (1) / *C. sartor* (1)	1.1
Cerambycidae	*Paracorymbia hybrida* (1) / *P. maculicornis* (1)	0.92
Elateridae	*Ampedus cardinalis* (3) / *A. praestus* (2) / *A. melonii* (1)	0
Elateridae	*Ampedus pomonae* (1) / *A. sanguinolentus* (1)	1.61
Elateridae	*Ampedus pomorum* (9) / *A. nemoralis* (3)	0
Lucanidae	*Lucanus cervus* (1) / *L. cervus fabiani* (1)	0
Nitidulidae	*Pityophagus ferrugineus* (1) / *P. laevior* (1)	1.88
Scarabaeidae	*Protaetia cuprea* (1) / *P. cuprea metallica* (1)	1.22
