# Supplementary material for: PASSIFOR: A reference library of DNA barcodes for French saproxylic beetles (Insecta, Coleoptera)
Source: Biodivers Data J. 2015 Mar 4;(3):e4078. doi: 10.3897/BDJ.3.e4078 (PMC4355675; doi:10.3897/BDJ.3.e4078)
Supplement: Supplementary material 3 — Neighbour Joining tree reconstructed from the 656 DNA barcodes of the PASSIFOR library. [file biodiversity_data_journal-3-e4078-s003.pdf]

BOLD TaxonID Tree

Title	:	PASSIFOR project - Reference library [DS-PSFOR01]
Date	:	4-August-2014
Data Type	:	Nucleotide
Distance Model	:	Kimura 2 Parameter
Marker	:	COI-5P
Codon Positions	:	1st, 2nd, 3rd
Labels	:	Country & Province, SampleID, Sequence Length, Family
Filters	:	Length > 200
Colorization	:	[blue]=Stop Codons [red]=Contamination or misidentification

Sequence Count	:	656
Species count	:	410
Genus count	:	251
Family count	:	40
Unidentified	:	0

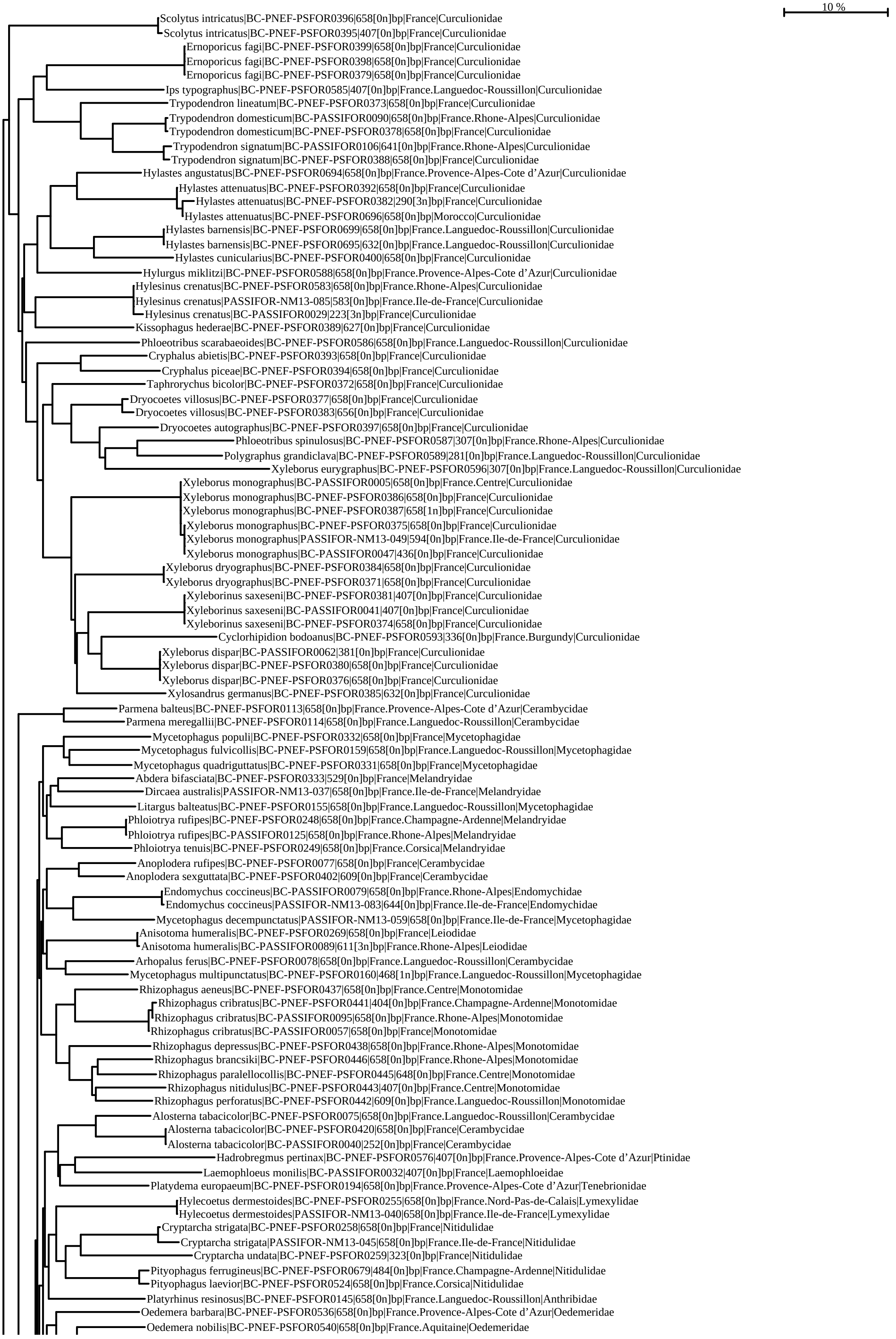
10 %
Scolytus intricatus|BC-PNEF-PSFOR0396|658[0n]bp|France|Curculionidae
Scolytus intricatus|BC-PNEF-PSFOR0395|407[0n]bp|France|Curculionidae
Ernoporicus fagi|BC-PNEF-PSFOR0399|658[0n]bp|France|Curculionidae
Ernoporicus fagi|BC-PNEF-PSFOR0398|658[0n]bp|France|Curculionidae
Ernoporicus fagi|BC-PNEF-PSFOR0379|658[0n]bp|France|Curculionidae
Ips typographus|BC-PNEF-PSFOR0585|407[0n]bp|France.Languedoc-Roussillon|Curculionidae
Trypodendron lineatum|BC-PNEF-PSFOR0373|658[0n]bp|France|Curculionidae
Trypodendron domesticum|BC-PASSIFOR0090|658[0n]bp|France.Rhone-Alpes|Curculionidae
Trypodendron domesticum|BC-PNEF-PSFOR0378|658[0n]bp|France|Curculionidae
Trypodendron signatum|BC-PASSIFOR0106|641[0n]bp|France.Rhone-Alpes|Curculionidae
Trypodendron signatum|BC-PNEF-PSFOR0388|658[0n]bp|France|Curculionidae
Hylastes angustatus|BC-PNEF-PSFOR0694|658[0n]bp|France.Provence-Alpes-Cote d'Azur|Curculionidae
Hylastes attenuatus|BC-PNEF-PSFOR0392|658[0n]bp|France|Curculionidae
Hylastes attenuatus|BC-PNEF-PSFOR0382|290[3n]bp|France|Curculionidae
Hylastes attenuatus|BC-PNEF-PSFOR0696|658[0n]bp|Morocco|Curculionidae
Hylastes barnensis|BC-PNEF-PSFOR0699|658[0n]bp|France.Languedoc-Roussillon|Curculionidae
Hylastes barnensis|BC-PNEF-PSFOR0695|632[0n]bp|France.Languedoc-Roussillon|Curculionidae
Hylastes cunicularius|BC-PNEF-PSFOR0400|658[0n]bp|France|Curculionidae
Hylurgus miklitzi|BC-PNEF-PSFOR0588|658[0n]bp|France.Provence-Alpes-Cote d'Azur|Curculionidae
Hylesinus crenatus|BC-PNEF-PSFOR0583|658[0n]bp|France.Rhone-Alpes|Curculionidae
Hylesinus crenatus|PASSIFOR-NM13-085|583[0n]bp|France.Ile-de-France|Curculionidae
Hylesinus crenatus|BC-PASSIFOR0029|223[3n]bp|France|Curculionidae
Kissophagus hederae|BC-PNEF-PSFOR0389|627[0n]bp|France|Curculionidae
Phloeotribus scarabaeoides|BC-PNEF-PSFOR0586|658[0n]bp|France.Languedoc-Roussillon|Curculionidae
Cryphalus abietis|BC-PNEF-PSFOR0393|658[0n]bp|France|Curculionidae
Cryphalus piceae|BC-PNEF-PSFOR0394|658[0n]bp|France|Curculionidae
Taphrorychus bicolor|BC-PNEF-PSFOR0372|658[0n]bp|France|Curculionidae
Dryocoetes villosus|BC-PNEF-PSFOR0377|658[0n]bp|France|Curculionidae
Dryocoetes villosus|BC-PNEF-PSFOR0383|656[0n]bp|France|Curculionidae
Dryocoetes autographus|BC-PNEF-PSFOR0397|658[0n]bp|France|Curculionidae
Phloeotribus spinulosus|BC-PNEF-PSFOR0587|307[0n]bp|France.Rhone-Alpes|Curculionidae
Polygraphus grandiclava|BC-PNEF-PSFOR0589|281[0n]bp|France.Languedoc-Roussillon|Curculionidae
Xyleborus eurygraphus|BC-PNEF-PSFOR0596|307[0n]bp|France.Languedoc-Roussillon|Curculionidae
Xyleborus monographus|BC-PASSIFOR0005|658[0n]bp|France.Centre|Curculionidae
Xyleborus monographus|BC-PNEF-PSFOR0386|658[0n]bp|France|Curculionidae
Xyleborus monographus|BC-PNEF-PSFOR0387|658[1n]bp|France|Curculionidae
Xyleborus monographus|BC-PNEF-PSFOR0375|658[0n]bp|France|Curculionidae
Xyleborus monographus|PASSIFOR-NM13-049|594[0n]bp|France.Ile-de-France|Curculionidae
Xyleborus monographus|BC-PASSIFOR0047|436[0n]bp|France|Curculionidae
Xyleborus dryographus|BC-PNEF-PSFOR0384|658[0n]bp|France|Curculionidae
Xyleborus dryographus|BC-PNEF-PSFOR0371|658[0n]bp|France|Curculionidae
Xyleborinus saxeseni|BC-PNEF-PSFOR0381|407[0n]bp|France|Curculionidae
Xyleborinus saxeseni|BC-PASSIFOR0041|407[0n]bp|France|Curculionidae
Xyleborinus saxeseni|BC-PNEF-PSFOR0374|658[0n]bp|France|Curculionidae
Cyclorhipidion bodoanus|BC-PNEF-PSFOR0593|336[0n]bp|France.Burgundy|Curculionidae
Xyleborus dispar|BC-PASSIFOR0062|381[0n]bp|France|Curculionidae
Xyleborus dispar|BC-PNEF-PSFOR0380|658[0n]bp|France|Curculionidae
Xyleborus dispar|BC-PNEF-PSFOR0376|658[0n]bp|France|Curculionidae
Xylosandrus germanus|BC-PNEF-PSFOR0385|632[0n]bp|France|Curculionidae
Parmena balteus|BC-PNEF-PSFOR0113|658[0n]bp|France.Provence-Alpes-Cote d'Azur|Cerambycidae
Parmena meregallii|BC-PNEF-PSFOR0114|658[0n]bp|France.Languedoc-Roussillon|Cerambycidae
Mycetophagus populi|BC-PNEF-PSFOR0332|658[0n]bp|France|Mycetophagidae
Mycetophagus fulvicollis|BC-PNEF-PSFOR0159|658[0n]bp|France.Languedoc-Roussillon|Mycetophagidae
Mycetophagus quadriguttatus|BC-PNEF-PSFOR0331|658[0n]bp|France|Mycetophagidae
Abdera bifasciata|BC-PNEF-PSFOR0333|529[0n]bp|France|Melandryidae
Dircaea australis|PASSIFOR-NM13-037|658[0n]bp|France.Ile-de-France|Melandryidae
Litargus balteatus|BC-PNEF-PSFOR0155|658[0n]bp|France.Languedoc-Roussillon|Mycetophagidae
Phloiotrya rufipes|BC-PNEF-PSFOR0248|658[0n]bp|France.Champagne-Ardenne|Melandryidae
Phloiotrya rufipes|BC-PASSIFOR0125|658[0n]bp|France.Rhone-Alpes|Melandryidae
Phloiotrya tenuis|BC-PNEF-PSFOR0249|658[0n]bp|France.Corsica|Melandryidae
Anoplodera rufipes|BC-PNEF-PSFOR0077|658[0n]bp|France|Cerambycidae
Anoplodera sexguttata|BC-PNEF-PSFOR0402|609[0n]bp|France|Cerambycidae
Endomychus coccineus|BC-PASSIFOR0079|658[0n]bp|France.Rhone-Alpes|Endomychidae
Endomychus coccineus|PASSIFOR-NM13-083|644[0n]bp|France.Ile-de-France|Endomychidae
Mycetophagus decempunctatus|PASSIFOR-NM13-059|658[0n]bp|France.Ile-de-France|Mycetophagidae
Anisotoma humeralis|BC-PNEF-PSFOR0269|658[0n]bp|France|Leiodidae
Anisotoma humeralis|BC-PASSIFOR0089|611[3n]bp|France.Rhone-Alpes|Leiodidae
Arhopalus ferus|BC-PNEF-PSFOR0078|658[0n]bp|France.Languedoc-Roussillon|Cerambycidae
Mycetophagus multipunctatus|BC-PNEF-PSFOR0160|468[1n]bp|France.Languedoc-Roussillon|Mycetophagidae
Rhizophagus aeneus|BC-PNEF-PSFOR0437|658[0n]bp|France.Centre|Monotomidae
Rhizophagus cribratus|BC-PNEF-PSFOR0441|404[0n]bp|France.Champagne-Ardenne|Monotomidae
Rhizophagus cribratus|BC-PASSIFOR0095|658[0n]bp|France.Rhone-Alpes|Monotomidae
Rhizophagus cribratus|BC-PASSIFOR0057|658[0n]bp|France|Monotomidae
Rhizophagus depressus|BC-PNEF-PSFOR0438|658[0n]bp|France.Rhone-Alpes|Monotomidae
Rhizophagus brancsiki|BC-PNEF-PSFOR0446|658[0n]bp|France.Rhone-Alpes|Monotomidae
Rhizophagus paralellocollis|BC-PNEF-PSFOR0445|648[0n]bp|France.Centre|Monotomidae
Rhizophagus nitidulus|BC-PNEF-PSFOR0443|407[0n]bp|France.Centre|Monotomidae
Rhizophagus perforatus|BC-PNEF-PSFOR0442|609[0n]bp|France.Languedoc-Roussillon|Monotomidae
Alosterna tabacicolor|BC-PNEF-PSFOR0075|658[0n]bp|France.Languedoc-Roussillon|Cerambycidae
Alosterna tabacicolor|BC-PNEF-PSFOR0420|658[0n]bp|France|Cerambycidae
Alosterna tabacicolor|BC-PASSIFOR0040|252[0n]bp|France|Cerambycidae
Hadrobregmus pertinax|BC-PNEF-PSFOR0576|407[0n]bp|France.Provence-Alpes-Cote d'Azur|Ptinidae
Laemophloeus monilis|BC-PASSIFOR0032|407[0n]bp|France|Laemophloeidae
Platydema europaeum|BC-PNEF-PSFOR0194|658[0n]bp|France.Provence-Alpes-Cote d'Azur|Tenebrionidae
Hylecoetus dermestoides|BC-PNEF-PSFOR0255|658[0n]bp|France.Nord-Pas-de-Calais|Lymexylidae
Hylecoetus dermestoides|PASSIFOR-NM13-040|658[0n]bp|France.Ile-de-France|Lymexylidae
Cryptarcha strigata|BC-PNEF-PSFOR0258|658[0n]bp|France|Nitidulidae
Cryptarcha strigata|PASSIFOR-NM13-045|658[0n]bp|France.Ile-de-France|Nitidulidae
Cryptarcha undata|BC-PNEF-PSFOR0259|323[0n]bp|France|Nitidulidae
Pityophagus ferrugineus|BC-PNEF-PSFOR0679|484[0n]bp|France.Champagne-Ardenne|Nitidulidae
Pityophagus laevior|BC-PNEF-PSFOR0524|658[0n]bp|France.Corsica|Nitidulidae
Platyrhinus resinosus|BC-PNEF-PSFOR0145|658[0n]bp|France.Languedoc-Roussillon|Anthribidae
Oedemera barbara|BC-PNEF-PSFOR0536|658[0n]bp|France.Provence-Alpes-Cote d'Azur|Oedemeridae
Oedemera nobilis|BC-PNEF-PSFOR0540|658[0n]bp|France.Aquitaine|Oedemeridae

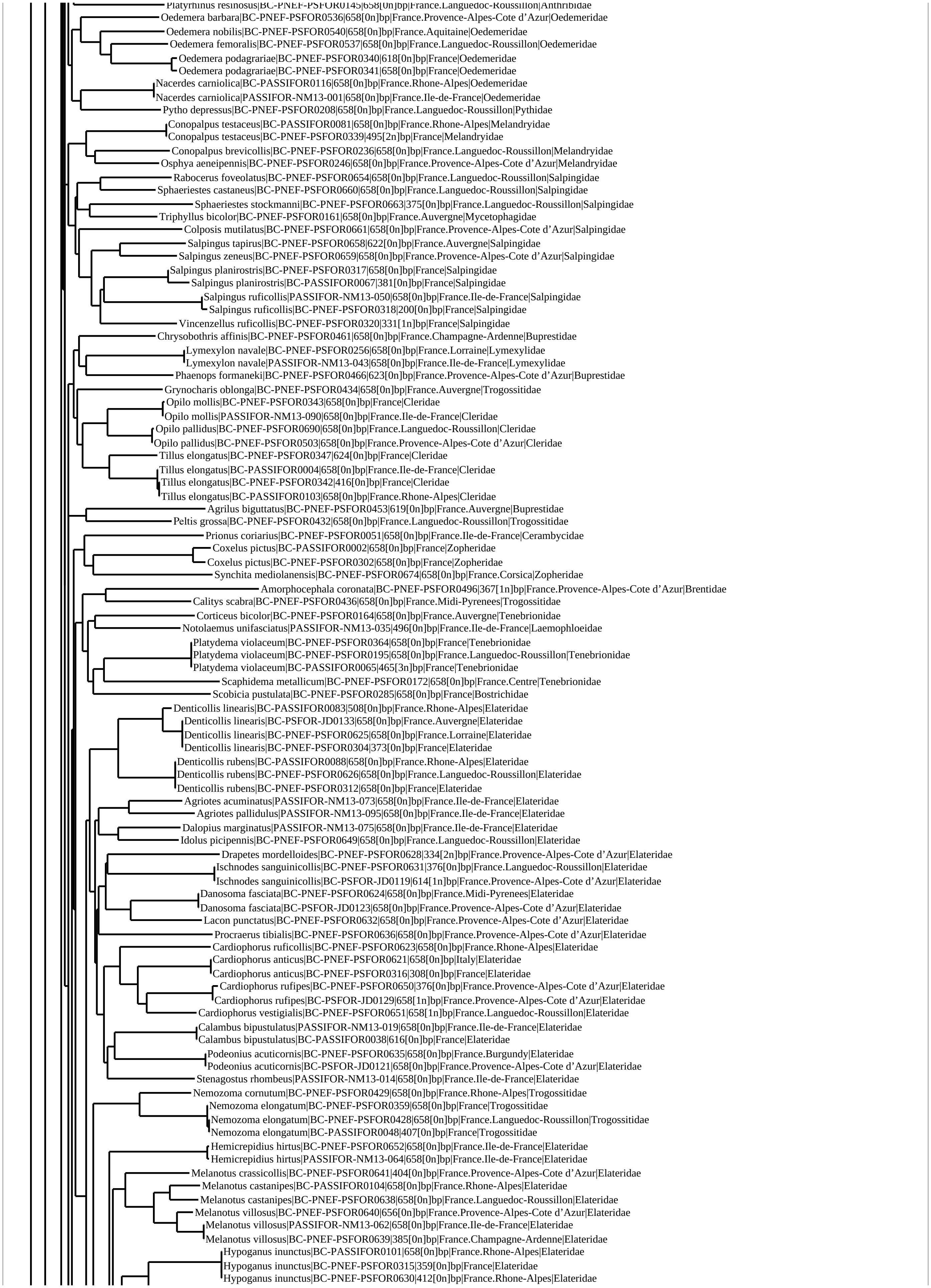
Platyrhinus resinosus|BC-PNEF-PSFOR0145|658[0n]bp|France.Languedoc-Roussillon|Anthribidae
Oedemera barbara|BC-PNEF-PSFOR0536|658[0n]bp|France.Provence-Alpes-Cote d'Azur|Oedemeridae
Oedemera nobilis|BC-PNEF-PSFOR0540|658[0n]bp|France.Aquitaine|Oedemeridae
Oedemera femoralis|BC-PNEF-PSFOR0537|658[0n]bp|France.Languedoc-Roussillon|Oedemeridae
Oedemera podagrariae|BC-PNEF-PSFOR0340|618[0n]bp|France|Oedemeridae
Oedemera podagrariae|BC-PNEF-PSFOR0341|658[0n]bp|France|Oedemeridae
Nacerdes carniolica|BC-PASSIFOR0116|658[0n]bp|France.Rhone-Alpes|Oedemeridae
Nacerdes carniolica|PASSIFOR-NM13-001|658[0n]bp|France.Ile-de-France|Oedemeridae
Pytho depressus|BC-PNEF-PSFOR0208|658[0n]bp|France.Languedoc-Roussillon|Pythidae
Conopalpus testaceus|BC-PASSIFOR0081|658[0n]bp|France.Rhone-Alpes|Melandryidae
Conopalpus testaceus|BC-PNEF-PSFOR0339|495[2n]bp|France|Melandryidae
Conopalpus brevicollis|BC-PNEF-PSFOR0236|658[0n]bp|France.Languedoc-Roussillon|Melandryidae
Osphya aeneipennis|BC-PNEF-PSFOR0246|658[0n]bp|France.Provence-Alpes-Cote d'Azur|Melandryidae
Rabocerus foveolatus|BC-PNEF-PSFOR0654|658[0n]bp|France.Languedoc-Roussillon|Salpingidae
Sphaeriestes castaneus|BC-PNEF-PSFOR0660|658[0n]bp|France.Languedoc-Roussillon|Salpingidae
Sphaeriestes stockmanni|BC-PNEF-PSFOR0663|375[0n]bp|France.Languedoc-Roussillon|Salpingidae
Triphyllus bicolor|BC-PNEF-PSFOR0161|658[0n]bp|France.Auvergne|Mycetophagidae
Colposis mutilatus|BC-PNEF-PSFOR0661|658[0n]bp|France.Provence-Alpes-Cote d'Azur|Salpingidae
Salpingus tapirus|BC-PNEF-PSFOR0658|622[0n]bp|France.Auvergne|Salpingidae
Salpingus zeneus|BC-PNEF-PSFOR0659|658[0n]bp|France.Provence-Alpes-Cote d'Azur|Salpingidae
Salpingus planirostris|BC-PNEF-PSFOR0317|658[0n]bp|France|Salpingidae
Salpingus planirostris|BC-PASSIFOR0067|381[0n]bp|France|Salpingidae
Salpingus ruficollis|PASSIFOR-NM13-050|658[0n]bp|France.Ile-de-France|Salpingidae
Salpingus ruficollis|BC-PNEF-PSFOR0318|200[0n]bp|France|Salpingidae
Vincenzellus ruficollis|BC-PNEF-PSFOR0320|331[1n]bp|France|Salpingidae
Chrysobothris affinis|BC-PNEF-PSFOR0461|658[0n]bp|France.Champagne-Ardenne|Buprestidae
Lymexylon navale|BC-PNEF-PSFOR0256|658[0n]bp|France.Lorraine|Lymexylidae
Lymexylon navale|PASSIFOR-NM13-043|658[0n]bp|France.Ile-de-France|Lymexylidae
Phaenops formaneki|BC-PNEF-PSFOR0466|623[0n]bp|France.Provence-Alpes-Cote d'Azur|Buprestidae
Grynocharis oblonga|BC-PNEF-PSFOR0434|658[0n]bp|France.Auvergne|Trogossitidae
Opilo mollis|BC-PNEF-PSFOR0343|658[0n]bp|France|Cleridae
Opilo mollis|PASSIFOR-NM13-090|658[0n]bp|France.Ile-de-France|Cleridae
Opilo pallidus|BC-PNEF-PSFOR0690|658[0n]bp|France.Languedoc-Roussillon|Cleridae
Opilo pallidus|BC-PNEF-PSFOR0503|658[0n]bp|France.Provence-Alpes-Cote d'Azur|Cleridae
Tillus elongatus|BC-PNEF-PSFOR0347|624[0n]bp|France|Cleridae
Tillus elongatus|BC-PASSIFOR0004|658[0n]bp|France.Ile-de-France|Cleridae
Tillus elongatus|BC-PNEF-PSFOR0342|416[0n]bp|France|Cleridae
Tillus elongatus|BC-PASSIFOR0103|658[0n]bp|France.Rhone-Alpes|Cleridae
Agrilus biguttatus|BC-PNEF-PSFOR0453|619[0n]bp|France.Auvergne|Buprestidae
Peltis grossa|BC-PNEF-PSFOR0432|658[0n]bp|France.Languedoc-Roussillon|Trogossitidae
Prionus coriarius|BC-PNEF-PSFOR0051|658[0n]bp|France.Ile-de-France|Cerambycidae
Coxelus pictus|BC-PASSIFOR0002|658[0n]bp|France|Zopheridae
Coxelus pictus|BC-PNEF-PSFOR0302|658[0n]bp|France|Zopheridae
Synchita mediolanensis|BC-PNEF-PSFOR0674|658[0n]bp|France.Corsica|Zopheridae
Amorphocephala coronata|BC-PNEF-PSFOR0496|367[1n]bp|France.Provence-Alpes-Cote d'Azur|Brentidae
Calitys scabra|BC-PNEF-PSFOR0436|658[0n]bp|France.Midi-Pyrenees|Trogossitidae
Corticeus bicolor|BC-PNEF-PSFOR0164|658[0n]bp|France.Auvergne|Tenebrionidae
Notolaemus unifasciatus|PASSIFOR-NM13-035|496[0n]bp|France.Ile-de-France|Laemophloeidae
Platydema violaceum|BC-PNEF-PSFOR0364|658[0n]bp|France|Tenebrionidae
Platydema violaceum|BC-PNEF-PSFOR0195|658[0n]bp|France.Languedoc-Roussillon|Tenebrionidae
Platydema violaceum|BC-PASSIFOR0065|465[3n]bp|France|Tenebrionidae
Scaphidema metallicum|BC-PNEF-PSFOR0172|658[0n]bp|France.Centre|Tenebrionidae
Scobicia pustulata|BC-PNEF-PSFOR0285|658[0n]bp|France|Bostrichidae
Denticollis linearis|BC-PASSIFOR0083|508[0n]bp|France.Rhone-Alpes|Elateridae
Denticollis linearis|BC-PSFOR-JD0133|658[0n]bp|France.Auvergne|Elateridae
Denticollis linearis|BC-PNEF-PSFOR0625|658[0n]bp|France.Lorraine|Elateridae
Denticollis linearis|BC-PNEF-PSFOR0304|373[0n]bp|France|Elateridae
Denticollis rubens|BC-PASSIFOR0088|658[0n]bp|France.Rhone-Alpes|Elateridae
Denticollis rubens|BC-PNEF-PSFOR0626|658[0n]bp|France.Languedoc-Roussillon|Elateridae
Denticollis rubens|BC-PNEF-PSFOR0312|658[0n]bp|France|Elateridae
Agriotes acuminatus|PASSIFOR-NM13-073|658[0n]bp|France.Ile-de-France|Elateridae
Agriotes pallidulus|PASSIFOR-NM13-095|658[0n]bp|France.Ile-de-France|Elateridae
Dalopius marginatus|PASSIFOR-NM13-075|658[0n]bp|France.Ile-de-France|Elateridae
Idolus picipennis|BC-PNEF-PSFOR0649|658[0n]bp|France.Languedoc-Roussillon|Elateridae
Drapetes mordelloides|BC-PNEF-PSFOR0628|334[2n]bp|France.Provence-Alpes-Cote d'Azur|Elateridae
Ischnodes sanguinicollis|BC-PNEF-PSFOR0631|376[0n]bp|France.Languedoc-Roussillon|Elateridae
Ischnodes sanguinicollis|BC-PSFOR-JD0119|614[1n]bp|France.Provence-Alpes-Cote d'Azur|Elateridae
Danosoma fasciata|BC-PNEF-PSFOR0624|658[0n]bp|France.Midi-Pyrenees|Elateridae
Danosoma fasciata|BC-PSFOR-JD0123|658[0n]bp|France.Provence-Alpes-Cote d'Azur|Elateridae
Lacon punctatus|BC-PNEF-PSFOR0632|658[0n]bp|France.Provence-Alpes-Cote d'Azur|Elateridae
Procraerus tibialis|BC-PNEF-PSFOR0636|658[0n]bp|France.Provence-Alpes-Cote d'Azur|Elateridae
Cardiophorus ruficollis|BC-PNEF-PSFOR0623|658[0n]bp|France.Rhone-Alpes|Elateridae
Cardiophorus anticus|BC-PNEF-PSFOR0621|658[0n]bp|Italy|Elateridae
Cardiophorus anticus|BC-PNEF-PSFOR0316|308[0n]bp|France|Elateridae
Cardiophorus rufipes|BC-PNEF-PSFOR0650|376[0n]bp|France.Provence-Alpes-Cote d'Azur|Elateridae
Cardiophorus rufipes|BC-PSFOR-JD0129|658[1n]bp|France.Provence-Alpes-Cote d'Azur|Elateridae
Cardiophorus vestigialis|BC-PNEF-PSFOR0651|658[1n]bp|France.Languedoc-Roussillon|Elateridae
Calambus bipustulatus|PASSIFOR-NM13-019|658[0n]bp|France.Ile-de-France|Elateridae
Calambus bipustulatus|BC-PASSIFOR0038|616[0n]bp|France|Elateridae
Podeonius acuticornis|BC-PNEF-PSFOR0635|658[0n]bp|France.Burgundy|Elateridae
Podeonius acuticornis|BC-PSFOR-JD0121|658[0n]bp|France.Provence-Alpes-Cote d'Azur|Elateridae
Stenagostus rhombeus|PASSIFOR-NM13-014|658[0n]bp|France.Ile-de-France|Elateridae
Nemozoma cornutum|BC-PNEF-PSFOR0429|658[0n]bp|France.Rhone-Alpes|Trogossitidae
Nemozoma elongatum|BC-PNEF-PSFOR0359|658[0n]bp|France|Trogossitidae
Nemozoma elongatum|BC-PNEF-PSFOR0428|658[0n]bp|France.Languedoc-Roussillon|Trogossitidae
Nemozoma elongatum|BC-PASSIFOR0048|407[0n]bp|France|Trogossitidae
Hemicrepidius hirtus|BC-PNEF-PSFOR0652|658[0n]bp|France.Ile-de-France|Elateridae
Hemicrepidius hirtus|PASSIFOR-NM13-064|658[0n]bp|France.Ile-de-France|Elateridae
Melanotus crassicollis|BC-PNEF-PSFOR0641|404[0n]bp|France.Provence-Alpes-Cote d'Azur|Elateridae
Melanotus castanipes|BC-PASSIFOR0104|658[0n]bp|France.Rhone-Alpes|Elateridae
Melanotus castanipes|BC-PNEF-PSFOR0638|658[0n]bp|France.Languedoc-Roussillon|Elateridae
Melanotus villosus|BC-PNEF-PSFOR0640|656[0n]bp|France.Provence-Alpes-Cote d'Azur|Elateridae
Melanotus villosus|PASSIFOR-NM13-062|658[0n]bp|France.Ile-de-France|Elateridae
Melanotus villosus|BC-PNEF-PSFOR0639|385[0n]bp|France.Champagne-Ardenne|Elateridae
Hypoganus inunctus|BC-PASSIFOR0101|658[0n]bp|France.Rhone-Alpes|Elateridae
Hypoganus inunctus|BC-PNEF-PSFOR0315|359[0n]bp|France|Elateridae
Hypoganus inunctus|BC-PNEF-PSFOR0630|412[0n]bp|France.Rhone-Alpes|Elateridae

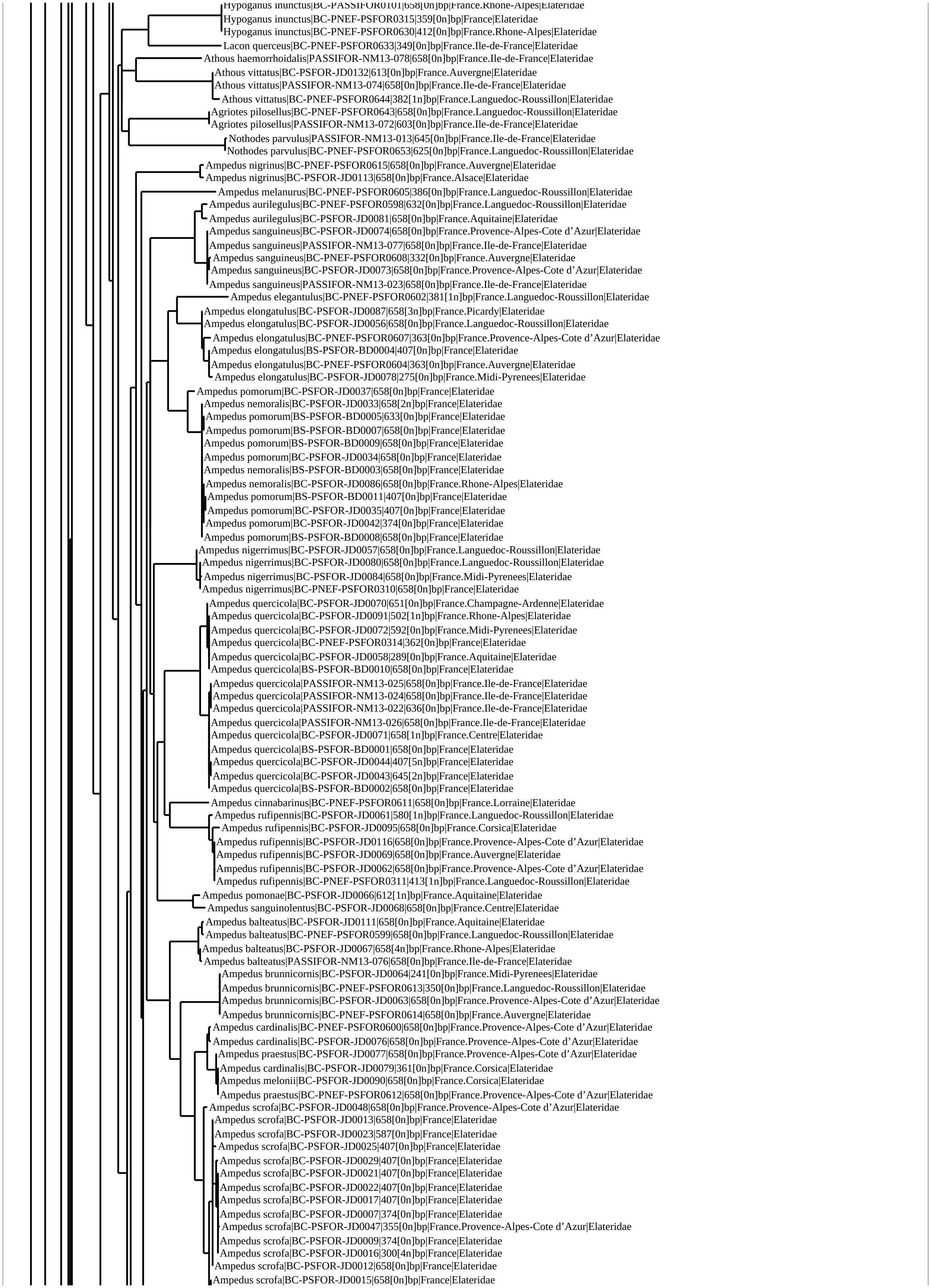
Hypoganus inunctus|BC-PASSIFOR0101|658[0n]bp|France.Rhone-Alpes|Elateridae
Hypoganus inunctus|BC-PNEF-PSFOR0315|359[0n]bp|France|Elateridae
Hypoganus inunctus|BC-PNEF-PSFOR0630|412[0n]bp|France.Rhone-Alpes|Elateridae
Lacon querceus|BC-PNEF-PSFOR0633|349[0n]bp|France.Ile-de-France|Elateridae
Athous haemorrhoidalis|PASSIFOR-NM13-078|658[0n]bp|France.Ile-de-France|Elateridae
Athous vittatus|BC-PSFOR-JD0132|613[0n]bp|France.Auvergne|Elateridae
Athous vittatus|PASSIFOR-NM13-074|658[0n]bp|France.Ile-de-France|Elateridae
Athous vittatus|BC-PNEF-PSFOR0644|382[1n]bp|France.Languedoc-Roussillon|Elateridae
Agriotes pilosellus|BC-PNEF-PSFOR0643|658[0n]bp|France.Languedoc-Roussillon|Elateridae
Agriotes pilosellus|PASSIFOR-NM13-072|603[0n]bp|France.Ile-de-France|Elateridae
Nothodes parvulus|PASSIFOR-NM13-013|645[0n]bp|France.Ile-de-France|Elateridae
Nothodes parvulus|BC-PNEF-PSFOR0653|625[0n]bp|France.Languedoc-Roussillon|Elateridae
Ampedus nigrinus|BC-PNEF-PSFOR0615|658[0n]bp|France.Auvergne|Elateridae
Ampedus nigrinus|BC-PSFOR-JD0113|658[0n]bp|France.Alsace|Elateridae
Ampedus melanurus|BC-PNEF-PSFOR0605|386[0n]bp|France.Languedoc-Roussillon|Elateridae
Ampedus aurilegulus|BC-PNEF-PSFOR0598|632[0n]bp|France.Languedoc-Roussillon|Elateridae
Ampedus aurilegulus|BC-PSFOR-JD0081|658[0n]bp|France.Aquitaine|Elateridae
Ampedus sanguineus|BC-PSFOR-JD0074|658[0n]bp|France.Provence-Alpes-Cote d’Azur|Elateridae
Ampedus sanguineus|PASSIFOR-NM13-077|658[0n]bp|France.Ile-de-France|Elateridae
Ampedus sanguineus|BC-PNEF-PSFOR0608|332[0n]bp|France.Auvergne|Elateridae
Ampedus sanguineus|BC-PSFOR-JD0073|658[0n]bp|France.Provence-Alpes-Cote d’Azur|Elateridae
Ampedus sanguineus|PASSIFOR-NM13-023|658[0n]bp|France.Ile-de-France|Elateridae
Ampedus elegantulus|BC-PNEF-PSFOR0602|381[1n]bp|France.Languedoc-Roussillon|Elateridae
Ampedus elongatulus|BC-PSFOR-JD0087|658[3n]bp|France.Picardy|Elateridae
Ampedus elongatulus|BC-PSFOR-JD0056|658[0n]bp|France.Languedoc-Roussillon|Elateridae
Ampedus elongatulus|BC-PNEF-PSFOR0607|363[0n]bp|France.Provence-Alpes-Cote d’Azur|Elateridae
Ampedus elongatulus|BS-PSFOR-BD0004|407[0n]bp|France|Elateridae
Ampedus elongatulus|BC-PNEF-PSFOR0604|363[0n]bp|France.Auvergne|Elateridae
Ampedus elongatulus|BC-PSFOR-JD0078|275[0n]bp|France.Midi-Pyrenees|Elateridae
Ampedus pomorum|BC-PSFOR-JD0037|658[0n]bp|France|Elateridae
Ampedus nemoralis|BC-PSFOR-JD0033|658[2n]bp|France|Elateridae
Ampedus pomorum|BS-PSFOR-BD0005|633[0n]bp|France|Elateridae
Ampedus pomorum|BS-PSFOR-BD0007|658[0n]bp|France|Elateridae
Ampedus pomorum|BS-PSFOR-BD0009|658[0n]bp|France|Elateridae
Ampedus pomorum|BC-PSFOR-JD0034|658[0n]bp|France|Elateridae
Ampedus nemoralis|BS-PSFOR-BD0003|658[0n]bp|France|Elateridae
Ampedus nemoralis|BC-PSFOR-JD0086|658[0n]bp|France.Rhone-Alpes|Elateridae
Ampedus pomorum|BS-PSFOR-BD0011|407[0n]bp|France|Elateridae
Ampedus pomorum|BC-PSFOR-JD0035|407[0n]bp|France|Elateridae
Ampedus pomorum|BC-PSFOR-JD0042|374[0n]bp|France|Elateridae
Ampedus pomorum|BS-PSFOR-BD0008|658[0n]bp|France|Elateridae
Ampedus nigerrimus|BC-PSFOR-JD0057|658[0n]bp|France.Languedoc-Roussillon|Elateridae
Ampedus nigerrimus|BC-PSFOR-JD0080|658[0n]bp|France.Languedoc-Roussillon|Elateridae
Ampedus nigerrimus|BC-PSFOR-JD0084|658[0n]bp|France.Midi-Pyrenees|Elateridae
Ampedus nigerrimus|BC-PNEF-PSFOR0310|658[0n]bp|France|Elateridae
Ampedus quercicola|BC-PSFOR-JD0070|651[0n]bp|France.Champagne-Ardenne|Elateridae
Ampedus quercicola|BC-PSFOR-JD0091|502[1n]bp|France.Rhone-Alpes|Elateridae
Ampedus quercicola|BC-PSFOR-JD0072|592[0n]bp|France.Midi-Pyrenees|Elateridae
Ampedus quercicola|BC-PNEF-PSFOR0314|362[0n]bp|France|Elateridae
Ampedus quercicola|BC-PSFOR-JD0058|289[0n]bp|France.Aquitaine|Elateridae
Ampedus quercicola|BS-PSFOR-BD0010|658[0n]bp|France|Elateridae
Ampedus quercicola|PASSIFOR-NM13-025|658[0n]bp|France.Ile-de-France|Elateridae
Ampedus quercicola|PASSIFOR-NM13-024|658[0n]bp|France.Ile-de-France|Elateridae
Ampedus quercicola|PASSIFOR-NM13-022|636[0n]bp|France.Ile-de-France|Elateridae
Ampedus quercicola|PASSIFOR-NM13-026|658[0n]bp|France.Ile-de-France|Elateridae
Ampedus quercicola|BC-PSFOR-JD0071|658[1n]bp|France.Centre|Elateridae
Ampedus quercicola|BS-PSFOR-BD0001|658[0n]bp|France|Elateridae
Ampedus quercicola|BC-PSFOR-JD0044|407[5n]bp|France|Elateridae
Ampedus quercicola|BC-PSFOR-JD0043|645[2n]bp|France|Elateridae
Ampedus quercicola|BS-PSFOR-BD0002|658[0n]bp|France|Elateridae
Ampedus cinnabarinus|BC-PNEF-PSFOR0611|658[0n]bp|France.Lorraine|Elateridae
Ampedus rufipennis|BC-PSFOR-JD0061|580[1n]bp|France.Languedoc-Roussillon|Elateridae
Ampedus rufipennis|BC-PSFOR-JD0095|658[0n]bp|France.Corsica|Elateridae
Ampedus rufipennis|BC-PSFOR-JD0116|658[0n]bp|France.Provence-Alpes-Cote d’Azur|Elateridae
Ampedus rufipennis|BC-PSFOR-JD0069|658[0n]bp|France.Auvergne|Elateridae
Ampedus rufipennis|BC-PSFOR-JD0062|658[0n]bp|France.Provence-Alpes-Cote d’Azur|Elateridae
Ampedus rufipennis|BC-PNEF-PSFOR0311|413[1n]bp|France.Languedoc-Roussillon|Elateridae
Ampedus pomonae|BC-PSFOR-JD0066|612[1n]bp|France.Aquitaine|Elateridae
Ampedus sanguinolentus|BC-PSFOR-JD0068|658[0n]bp|France.Centre|Elateridae
Ampedus balteatus|BC-PSFOR-JD0111|658[0n]bp|France.Aquitaine|Elateridae
Ampedus balteatus|BC-PNEF-PSFOR0599|658[0n]bp|France.Languedoc-Roussillon|Elateridae
Ampedus balteatus|BC-PSFOR-JD0067|658[4n]bp|France.Rhone-Alpes|Elateridae
Ampedus balteatus|PASSIFOR-NM13-076|658[0n]bp|France.Ile-de-France|Elateridae
Ampedus brunnicornis|BC-PSFOR-JD0064|241[0n]bp|France.Midi-Pyrenees|Elateridae
Ampedus brunnicornis|BC-PNEF-PSFOR0613|350[0n]bp|France.Languedoc-Roussillon|Elateridae
Ampedus brunnicornis|BC-PSFOR-JD0063|658[0n]bp|France.Provence-Alpes-Cote d’Azur|Elateridae
Ampedus brunnicornis|BC-PNEF-PSFOR0614|658[0n]bp|France.Auvergne|Elateridae
Ampedus cardinalis|BC-PNEF-PSFOR0600|658[0n]bp|France.Provence-Alpes-Cote d’Azur|Elateridae
Ampedus cardinalis|BC-PSFOR-JD0076|658[0n]bp|France.Provence-Alpes-Cote d’Azur|Elateridae
Ampedus praestus|BC-PSFOR-JD0077|658[0n]bp|France.Provence-Alpes-Cote d’Azur|Elateridae
Ampedus cardinalis|BC-PSFOR-JD0079|361[0n]bp|France.Corsica|Elateridae
Ampedus melonii|BC-PSFOR-JD0090|658[0n]bp|France.Corsica|Elateridae
Ampedus praestus|BC-PNEF-PSFOR0612|658[0n]bp|France.Provence-Alpes-Cote d’Azur|Elateridae
Ampedus scrofa|BC-PSFOR-JD0048|658[0n]bp|France.Provence-Alpes-Cote d’Azur|Elateridae
Ampedus scrofa|BC-PSFOR-JD0013|658[0n]bp|France|Elateridae
Ampedus scrofa|BC-PSFOR-JD0023|587[0n]bp|France|Elateridae
Ampedus scrofa|BC-PSFOR-JD0025|407[0n]bp|France|Elateridae
Ampedus scrofa|BC-PSFOR-JD0029|407[0n]bp|France|Elateridae
Ampedus scrofa|BC-PSFOR-JD0021|407[0n]bp|France|Elateridae
Ampedus scrofa|BC-PSFOR-JD0022|407[0n]bp|France|Elateridae
Ampedus scrofa|BC-PSFOR-JD0017|407[0n]bp|France|Elateridae
Ampedus scrofa|BC-PSFOR-JD0007|374[0n]bp|France|Elateridae
Ampedus scrofa|BC-PSFOR-JD0047|355[0n]bp|France.Provence-Alpes-Cote d’Azur|Elateridae
Ampedus scrofa|BC-PSFOR-JD0009|374[0n]bp|France|Elateridae
Ampedus scrofa|BC-PSFOR-JD0016|300[4n]bp|France|Elateridae
Ampedus scrofa|BC-PSFOR-JD0012|658[0n]bp|France|Elateridae
Ampedus scrofa|BC-PSFOR-JD0015|658[0n]bp|France|Elateridae

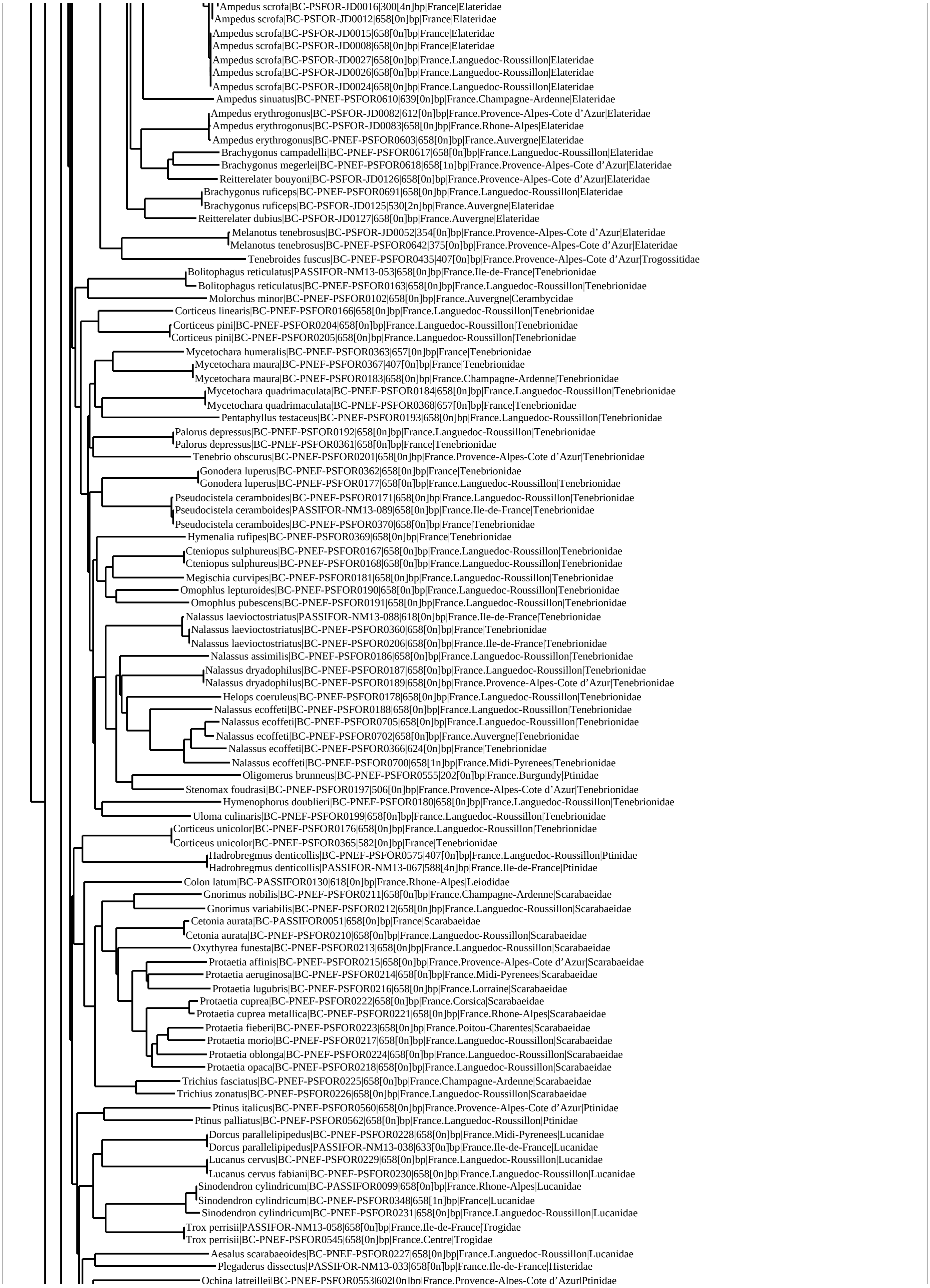
Ampedus scrofa|BC-PSFOR-JD0016|300[4n]bp|France|Elateridae
Ampedus scrofa|BC-PSFOR-JD0012|658[0n]bp|France|Elateridae
Ampedus scrofa|BC-PSFOR-JD0015|658[0n]bp|France|Elateridae
Ampedus scrofa|BC-PSFOR-JD0008|658[0n]bp|France|Elateridae
Ampedus scrofa|BC-PSFOR-JD0027|658[0n]bp|France.Languedoc-Roussillon|Elateridae
Ampedus scrofa|BC-PSFOR-JD0026|658[0n]bp|France.Languedoc-Roussillon|Elateridae
Ampedus scrofa|BC-PSFOR-JD0024|658[0n]bp|France.Languedoc-Roussillon|Elateridae
Ampedus sinuatus|BC-PNEF-PSFOR0610|639[0n]bp|France.Champagne-Ardenne|Elateridae
Ampedus erythrogonus|BC-PSFOR-JD0082|612[0n]bp|France.Provence-Alpes-Cote d'Azur|Elateridae
Ampedus erythrogonus|BC-PSFOR-JD0083|658[0n]bp|France.Rhone-Alpes|Elateridae
Ampedus erythrogonus|BC-PNEF-PSFOR0603|658[0n]bp|France.Auvergne|Elateridae
Brachygonus campadelli|BC-PNEF-PSFOR0617|658[0n]bp|France.Languedoc-Roussillon|Elateridae
Brachygonus megerlei|BC-PNEF-PSFOR0618|658[1n]bp|France.Provence-Alpes-Cote d'Azur|Elateridae
Reitterelater bouyoni|BC-PSFOR-JD0126|658[0n]bp|France.Provence-Alpes-Cote d'Azur|Elateridae
Brachygonus ruficeps|BC-PNEF-PSFOR0691|658[0n]bp|France.Languedoc-Roussillon|Elateridae
Brachygonus ruficeps|BC-PSFOR-JD0125|530[2n]bp|France.Auvergne|Elateridae
Reitterelater dubius|BC-PSFOR-JD0127|658[0n]bp|France.Auvergne|Elateridae
Melanotus tenebrosus|BC-PSFOR-JD0052|354[0n]bp|France.Provence-Alpes-Cote d'Azur|Elateridae
Melanotus tenebrosus|BC-PNEF-PSFOR0642|375[0n]bp|France.Provence-Alpes-Cote d'Azur|Elateridae
Tenebroides fuscus|BC-PNEF-PSFOR0435|407[0n]bp|France.Provence-Alpes-Cote d'Azur|Trogossitidae
Bolitophagus reticulatus|PASSIFOR-NM13-053|658[0n]bp|France.Ile-de-France|Tenebrionidae
Bolitophagus reticulatus|BC-PNEF-PSFOR0163|658[0n]bp|France.Languedoc-Roussillon|Tenebrionidae
Molorchus minor|BC-PNEF-PSFOR0102|658[0n]bp|France.Auvergne|Cerambycidae
Corticeus linearis|BC-PNEF-PSFOR0166|658[0n]bp|France.Languedoc-Roussillon|Tenebrionidae
Corticeus pini|BC-PNEF-PSFOR0204|658[0n]bp|France.Languedoc-Roussillon|Tenebrionidae
Corticeus pini|BC-PNEF-PSFOR0205|658[0n]bp|France.Languedoc-Roussillon|Tenebrionidae
Mycetochara humeralis|BC-PNEF-PSFOR0363|657[0n]bp|France|Tenebrionidae
Mycetochara maura|BC-PNEF-PSFOR0367|407[0n]bp|France|Tenebrionidae
Mycetochara maura|BC-PNEF-PSFOR0183|658[0n]bp|France.Champagne-Ardenne|Tenebrionidae
Mycetochara quadrimaculata|BC-PNEF-PSFOR0184|658[0n]bp|France.Languedoc-Roussillon|Tenebrionidae
Mycetochara quadrimaculata|BC-PNEF-PSFOR0368|657[0n]bp|France|Tenebrionidae
Pentaphyllus testaceus|BC-PNEF-PSFOR0193|658[0n]bp|France.Languedoc-Roussillon|Tenebrionidae
Palorus depressus|BC-PNEF-PSFOR0192|658[0n]bp|France.Languedoc-Roussillon|Tenebrionidae
Palorus depressus|BC-PNEF-PSFOR0361|658[0n]bp|France|Tenebrionidae
Tenebrio obscurus|BC-PNEF-PSFOR0201|658[0n]bp|France.Provence-Alpes-Cote d'Azur|Tenebrionidae
Gonodera luperus|BC-PNEF-PSFOR0362|658[0n]bp|France|Tenebrionidae
Gonodera luperus|BC-PNEF-PSFOR0177|658[0n]bp|France.Languedoc-Roussillon|Tenebrionidae
Pseudocistela ceramboides|BC-PNEF-PSFOR0171|658[0n]bp|France.Languedoc-Roussillon|Tenebrionidae
Pseudocistela ceramboides|PASSIFOR-NM13-089|658[0n]bp|France.Ile-de-France|Tenebrionidae
Pseudocistela ceramboides|BC-PNEF-PSFOR0370|658[0n]bp|France|Tenebrionidae
Hymenalia rufipes|BC-PNEF-PSFOR0369|658[0n]bp|France|Tenebrionidae
Cteniopus sulphureus|BC-PNEF-PSFOR0167|658[0n]bp|France.Languedoc-Roussillon|Tenebrionidae
Cteniopus sulphureus|BC-PNEF-PSFOR0168|658[0n]bp|France.Languedoc-Roussillon|Tenebrionidae
Megischia curvipes|BC-PNEF-PSFOR0181|658[0n]bp|France.Languedoc-Roussillon|Tenebrionidae
Omophlus lepturoides|BC-PNEF-PSFOR0190|658[0n]bp|France.Languedoc-Roussillon|Tenebrionidae
Omophlus pubescens|BC-PNEF-PSFOR0191|658[0n]bp|France.Languedoc-Roussillon|Tenebrionidae
Nalassus laevioctostriatus|PASSIFOR-NM13-088|618[0n]bp|France.Ile-de-France|Tenebrionidae
Nalassus laevioctostriatus|BC-PNEF-PSFOR0360|658[0n]bp|France|Tenebrionidae
Nalassus laevioctostriatus|BC-PNEF-PSFOR0206|658[0n]bp|France.Ile-de-France|Tenebrionidae
Nalassus assimilis|BC-PNEF-PSFOR0186|658[0n]bp|France.Languedoc-Roussillon|Tenebrionidae
Nalassus dryadophilus|BC-PNEF-PSFOR0187|658[0n]bp|France.Languedoc-Roussillon|Tenebrionidae
Nalassus dryadophilus|BC-PNEF-PSFOR0189|658[0n]bp|France.Provence-Alpes-Cote d'Azur|Tenebrionidae
Helops coeruleus|BC-PNEF-PSFOR0178|658[0n]bp|France.Languedoc-Roussillon|Tenebrionidae
Nalassus ecoffeti|BC-PNEF-PSFOR0188|658[0n]bp|France.Languedoc-Roussillon|Tenebrionidae
Nalassus ecoffeti|BC-PNEF-PSFOR0705|658[0n]bp|France.Languedoc-Roussillon|Tenebrionidae
Nalassus ecoffeti|BC-PNEF-PSFOR0702|658[0n]bp|France.Auvergne|Tenebrionidae
Nalassus ecoffeti|BC-PNEF-PSFOR0366|624[0n]bp|France|Tenebrionidae
Nalassus ecoffeti|BC-PNEF-PSFOR0700|658[1n]bp|France.Midi-Pyrenees|Tenebrionidae
Oligomerus brunneus|BC-PNEF-PSFOR0555|202[0n]bp|France.Burgundy|Ptinidae
Stenomax foudrasi|BC-PNEF-PSFOR0197|506[0n]bp|France.Provence-Alpes-Cote d'Azur|Tenebrionidae
Hymenophorus doublieri|BC-PNEF-PSFOR0180|658[0n]bp|France.Languedoc-Roussillon|Tenebrionidae
Uloma culinaris|BC-PNEF-PSFOR0199|658[0n]bp|France.Languedoc-Roussillon|Tenebrionidae
Corticeus unicolor|BC-PNEF-PSFOR0176|658[0n]bp|France.Languedoc-Roussillon|Tenebrionidae
Corticeus unicolor|BC-PNEF-PSFOR0365|582[0n]bp|France|Tenebrionidae
Hadrobregmus denticollis|BC-PNEF-PSFOR0575|407[0n]bp|France.Languedoc-Roussillon|Ptinidae
Hadrobregmus denticollis|PASSIFOR-NM13-067|588[4n]bp|France.Ile-de-France|Ptinidae
Colon latum|BC-PASSIFOR0130|618[0n]bp|France.Rhone-Alpes|Leiodidae
Gnorimus nobilis|BC-PNEF-PSFOR0211|658[0n]bp|France.Champagne-Ardenne|Scarabaeidae
Gnorimus variabilis|BC-PNEF-PSFOR0212|658[0n]bp|France.Languedoc-Roussillon|Scarabaeidae
Cetonia aurata|BC-PASSIFOR0051|658[0n]bp|France|Scarabaeidae
Cetonia aurata|BC-PNEF-PSFOR0210|658[0n]bp|France.Languedoc-Roussillon|Scarabaeidae
Oxythyrea funesta|BC-PNEF-PSFOR0213|658[0n]bp|France.Languedoc-Roussillon|Scarabaeidae
Protaetia affinis|BC-PNEF-PSFOR0215|658[0n]bp|France.Provence-Alpes-Cote d'Azur|Scarabaeidae
Protaetia aeruginosa|BC-PNEF-PSFOR0214|658[0n]bp|France.Midi-Pyrenees|Scarabaeidae
Protaetia lugubris|BC-PNEF-PSFOR0216|658[0n]bp|France.Lorraine|Scarabaeidae
Protaetia cuprea|BC-PNEF-PSFOR0222|658[0n]bp|France.Corsica|Scarabaeidae
Protaetia cuprea metallica|BC-PNEF-PSFOR0221|658[0n]bp|France.Rhone-Alpes|Scarabaeidae
Protaetia fieberi|BC-PNEF-PSFOR0223|658[0n]bp|France.Poitou-Charentes|Scarabaeidae
Protaetia morio|BC-PNEF-PSFOR0217|658[0n]bp|France.Languedoc-Roussillon|Scarabaeidae
Protaetia oblonga|BC-PNEF-PSFOR0224|658[0n]bp|France.Languedoc-Roussillon|Scarabaeidae
Protaetia opaca|BC-PNEF-PSFOR0218|658[0n]bp|France.Languedoc-Roussillon|Scarabaeidae
Trichius fasciatus|BC-PNEF-PSFOR0225|658[0n]bp|France.Champagne-Ardenne|Scarabaeidae
Trichius zonatus|BC-PNEF-PSFOR0226|658[0n]bp|France.Languedoc-Roussillon|Scarabaeidae
Ptinus italicus|BC-PNEF-PSFOR0560|658[0n]bp|France.Provence-Alpes-Cote d'Azur|Ptinidae
Ptinus palliatus|BC-PNEF-PSFOR0562|658[0n]bp|France.Languedoc-Roussillon|Ptinidae
Dorcus parallelipipedus|BC-PNEF-PSFOR0228|658[0n]bp|France.Midi-Pyrenees|Lucanidae
Dorcus parallelipipedus|PASSIFOR-NM13-038|633[0n]bp|France.Ile-de-France|Lucanidae
Lucanus cervus|BC-PNEF-PSFOR0229|658[0n]bp|France.Languedoc-Roussillon|Lucanidae
Lucanus cervus fabiani|BC-PNEF-PSFOR0230|658[0n]bp|France.Languedoc-Roussillon|Lucanidae
Sinodendron cylindricum|BC-PASSIFOR0099|658[0n]bp|France.Rhone-Alpes|Lucanidae
Sinodendron cylindricum|BC-PNEF-PSFOR0348|658[1n]bp|France|Lucanidae
Sinodendron cylindricum|BC-PNEF-PSFOR0231|658[0n]bp|France.Languedoc-Roussillon|Lucanidae
Trox perrisii|PASSIFOR-NM13-058|658[0n]bp|France.Ile-de-France|Trogidae
Trox perrisii|BC-PNEF-PSFOR0545|658[0n]bp|France.Centre|Trogidae
Aesalus scarabaeoides|BC-PNEF-PSFOR0227|658[0n]bp|France.Languedoc-Roussillon|Lucanidae
Plegaderus dissectus|PASSIFOR-NM13-033|658[0n]bp|France.Ile-de-France|Histeridae
Ochina latreillei|BC-PNEF-PSFOR0553|602[0n]bp|France.Provence-Alpes-Cote d'Azur|Ptinidae

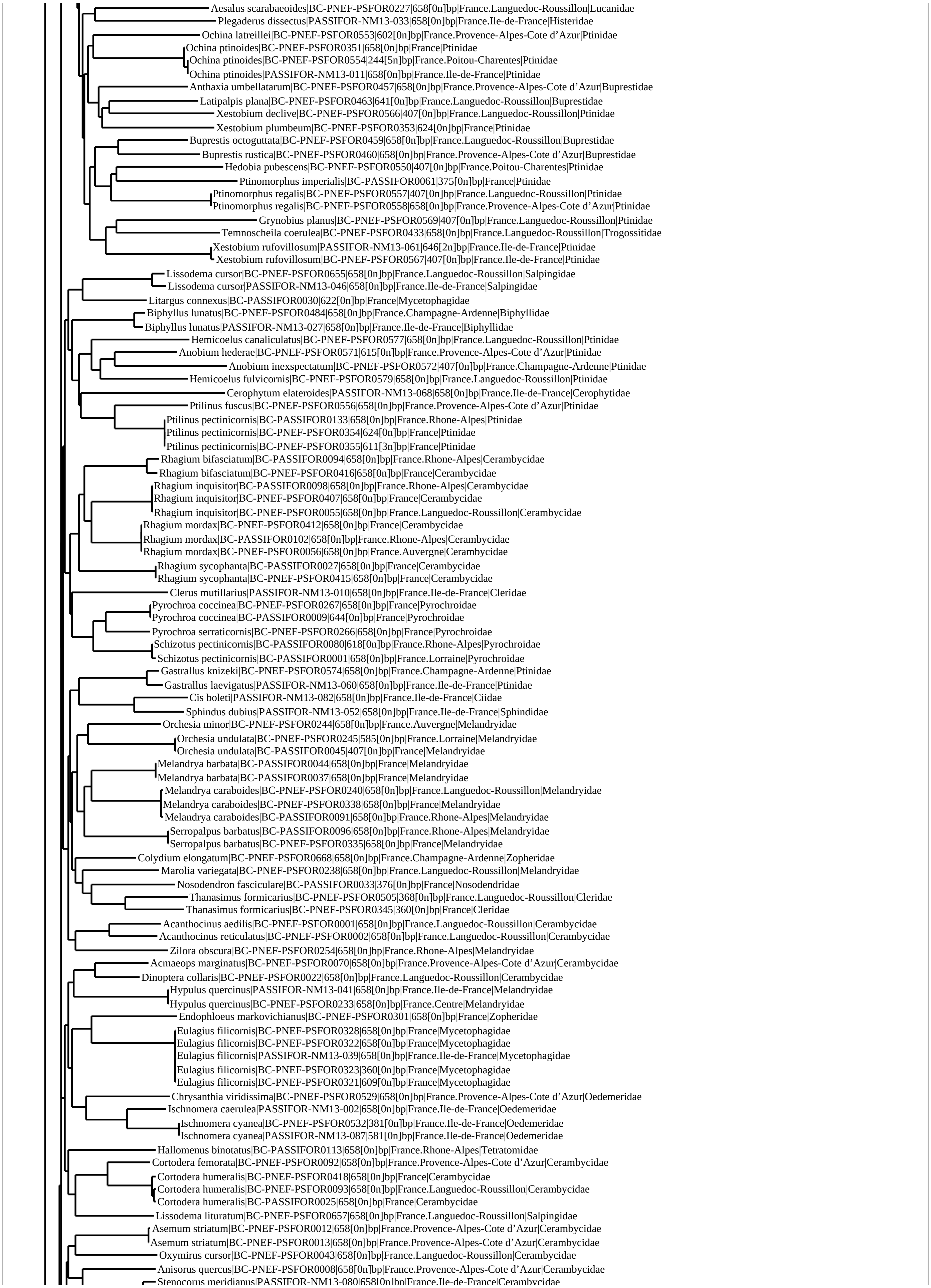
Aesalus scarabaeoides|BC-PNEF-PSFOR0227|658[0n]bp|France.Languedoc-Roussillon|Lucanidae
Plegaderus dissectus|PASSIFOR-NM13-033|658[0n]bp|France.Ile-de-France|Histeridae
Ochina latreillei|BC-PNEF-PSFOR0553|602[0n]bp|France.Provence-Alpes-Cote d'Azur|Ptinidae
Ochina ptinoides|BC-PNEF-PSFOR0351|658[0n]bp|France|Ptinidae
Ochina ptinoides|BC-PNEF-PSFOR0554|244[5n]bp|France.Poitou-Charentes|Ptinidae
Ochina ptinoides|PASSIFOR-NM13-011|658[0n]bp|France.Ile-de-France|Ptinidae
Anthaxia umbellatarum|BC-PNEF-PSFOR0457|658[0n]bp|France.Provence-Alpes-Cote d'Azur|Buprestidae
Latipalpis plana|BC-PNEF-PSFOR0463|641[0n]bp|France.Languedoc-Roussillon|Buprestidae
Xestobium declive|BC-PNEF-PSFOR0566|407[0n]bp|France.Languedoc-Roussillon|Ptinidae
Xestobium plumbeum|BC-PNEF-PSFOR0353|624[0n]bp|France|Ptinidae
Buprestis octoguttata|BC-PNEF-PSFOR0459|658[0n]bp|France.Languedoc-Roussillon|Buprestidae
Buprestis rustica|BC-PNEF-PSFOR0460|658[0n]bp|France.Provence-Alpes-Cote d'Azur|Buprestidae
Hedobia pubescens|BC-PNEF-PSFOR0550|407[0n]bp|France.Poitou-Charentes|Ptinidae
Ptinomorphus imperialis|BC-PASSIFOR0061|375[0n]bp|France|Ptinidae
Ptinomorphus regalis|BC-PNEF-PSFOR0557|407[0n]bp|France.Languedoc-Roussillon|Ptinidae
Ptinomorphus regalis|BC-PNEF-PSFOR0558|658[0n]bp|France.Provence-Alpes-Cote d'Azur|Ptinidae
Grynobius planus|BC-PNEF-PSFOR0569|407[0n]bp|France.Languedoc-Roussillon|Ptinidae
Temnoscheila coerulea|BC-PNEF-PSFOR0433|658[0n]bp|France.Languedoc-Roussillon|Trogossitidae
Xestobium rufovillosum|PASSIFOR-NM13-061|646[2n]bp|France.Ile-de-France|Ptinidae
Xestobium rufovillosum|BC-PNEF-PSFOR0567|407[0n]bp|France.Ile-de-France|Ptinidae
Lissodema cursor|BC-PNEF-PSFOR0655|658[0n]bp|France.Languedoc-Roussillon|Salpingidae
Lissodema cursor|PASSIFOR-NM13-046|658[0n]bp|France.Ile-de-France|Salpingidae
Litargus connexus|BC-PASSIFOR0030|622[0n]bp|France|Mycetophagidae
Biphyllus lunatus|BC-PNEF-PSFOR0484|658[0n]bp|France.Champagne-Ardenne|Biphyllidae
Biphyllus lunatus|PASSIFOR-NM13-027|658[0n]bp|France.Ile-de-France|Biphyllidae
Hemicoelus canaliculatus|BC-PNEF-PSFOR0577|658[0n]bp|France.Languedoc-Roussillon|Ptinidae
Anobium hederae|BC-PNEF-PSFOR0571|615[0n]bp|France.Provence-Alpes-Cote d'Azur|Ptinidae
Anobium inexspectatum|BC-PNEF-PSFOR0572|407[0n]bp|France.Champagne-Ardenne|Ptinidae
Hemicoelus fulvicornis|BC-PNEF-PSFOR0579|658[0n]bp|France.Languedoc-Roussillon|Ptinidae
Cerophytum elateroides|PASSIFOR-NM13-068|658[0n]bp|France.Ile-de-France|Cerophytidae
Ptilinus fuscus|BC-PNEF-PSFOR0556|658[0n]bp|France.Provence-Alpes-Cote d'Azur|Ptinidae
Ptilinus pectinicornis|BC-PASSIFOR0133|658[0n]bp|France.Rhone-Alpes|Ptinidae
Ptilinus pectinicornis|BC-PNEF-PSFOR0354|624[0n]bp|France|Ptinidae
Ptilinus pectinicornis|BC-PNEF-PSFOR0355|611[3n]bp|France|Ptinidae
Rhagium bifasciatum|BC-PASSIFOR0094|658[0n]bp|France.Rhone-Alpes|Cerambycidae
Rhagium bifasciatum|BC-PNEF-PSFOR0416|658[0n]bp|France|Cerambycidae
Rhagium inquisitor|BC-PASSIFOR0098|658[0n]bp|France.Rhone-Alpes|Cerambycidae
Rhagium inquisitor|BC-PNEF-PSFOR0407|658[0n]bp|France|Cerambycidae
Rhagium inquisitor|BC-PNEF-PSFOR0055|658[0n]bp|France.Languedoc-Roussillon|Cerambycidae
Rhagium mordax|BC-PNEF-PSFOR0412|658[0n]bp|France|Cerambycidae
Rhagium mordax|BC-PASSIFOR0102|658[0n]bp|France.Rhone-Alpes|Cerambycidae
Rhagium mordax|BC-PNEF-PSFOR0056|658[0n]bp|France.Auvergne|Cerambycidae
Rhagium sycophanta|BC-PASSIFOR0027|658[0n]bp|France|Cerambycidae
Rhagium sycophanta|BC-PNEF-PSFOR0415|658[0n]bp|France|Cerambycidae
Clerus mutillarius|PASSIFOR-NM13-010|658[0n]bp|France.Ile-de-France|Cleridae
Pyrochroa coccinea|BC-PNEF-PSFOR0267|658[0n]bp|France|Pyrochroidae
Pyrochroa coccinea|BC-PASSIFOR0009|644[0n]bp|France|Pyrochroidae
Pyrochroa serraticornis|BC-PNEF-PSFOR0266|658[0n]bp|France|Pyrochroidae
Schizotus pectinicornis|BC-PASSIFOR0080|618[0n]bp|France.Rhone-Alpes|Pyrochroidae
Schizotus pectinicornis|BC-PASSIFOR0001|658[0n]bp|France.Lorraine|Pyrochroidae
Gastrallus knizeki|BC-PNEF-PSFOR0574|658[0n]bp|France.Champagne-Ardenne|Ptinidae
Gastrallus laevigatus|PASSIFOR-NM13-060|658[0n]bp|France.Ile-de-France|Ptinidae
Cis boleti|PASSIFOR-NM13-082|658[0n]bp|France.Ile-de-France|Ciidae
Sphindus dubius|PASSIFOR-NM13-052|658[0n]bp|France.Ile-de-France|Sphindidae
Orchesia minor|BC-PNEF-PSFOR0244|658[0n]bp|France.Auvergne|Melandryidae
Orchesia undulata|BC-PNEF-PSFOR0245|585[0n]bp|France.Lorraine|Melandryidae
Orchesia undulata|BC-PASSIFOR0045|407[0n]bp|France|Melandryidae
Melandrya barbata|BC-PASSIFOR0044|658[0n]bp|France|Melandryidae
Melandrya barbata|BC-PASSIFOR0037|658[0n]bp|France|Melandryidae
Melandrya caraboides|BC-PNEF-PSFOR0240|658[0n]bp|France.Languedoc-Roussillon|Melandryidae
Melandrya caraboides|BC-PNEF-PSFOR0338|658[0n]bp|France|Melandryidae
Melandrya caraboides|BC-PASSIFOR0091|658[0n]bp|France.Rhone-Alpes|Melandryidae
Serropalpus barbatus|BC-PASSIFOR0096|658[0n]bp|France.Rhone-Alpes|Melandryidae
Serropalpus barbatus|BC-PNEF-PSFOR0335|658[0n]bp|France|Melandryidae
Colydium elongatum|BC-PNEF-PSFOR0668|658[0n]bp|France.Champagne-Ardenne|Zopheridae
Marolia variegata|BC-PNEF-PSFOR0238|658[0n]bp|France.Languedoc-Roussillon|Melandryidae
Nosodendron fasciculare|BC-PASSIFOR0033|376[0n]bp|France|Nosodendridae
Thanasimus formicarius|BC-PNEF-PSFOR0505|368[0n]bp|France.Languedoc-Roussillon|Cleridae
Thanasimus formicarius|BC-PNEF-PSFOR0345|360[0n]bp|France|Cleridae
Acanthocinus aedilis|BC-PNEF-PSFOR0001|658[0n]bp|France.Languedoc-Roussillon|Cerambycidae
Acanthocinus reticulatus|BC-PNEF-PSFOR0002|658[0n]bp|France.Languedoc-Roussillon|Cerambycidae
Zilora obscura|BC-PNEF-PSFOR0254|658[0n]bp|France.Rhone-Alpes|Melandryidae
Acmaeops marginatus|BC-PNEF-PSFOR0070|658[0n]bp|France.Provence-Alpes-Cote d'Azur|Cerambycidae
Dinoptera collaris|BC-PNEF-PSFOR0022|658[0n]bp|France.Languedoc-Roussillon|Cerambycidae
Hypulus quercinus|PASSIFOR-NM13-041|658[0n]bp|France.Ile-de-France|Melandryidae
Hypulus quercinus|BC-PNEF-PSFOR0233|658[0n]bp|France.Centre|Melandryidae
Endophloeus markovichianus|BC-PNEF-PSFOR0301|658[0n]bp|France|Zopheridae
Eulagius filicornis|BC-PNEF-PSFOR0328|658[0n]bp|France|Mycetophagidae
Eulagius filicornis|BC-PNEF-PSFOR0322|658[0n]bp|France|Mycetophagidae
Eulagius filicornis|PASSIFOR-NM13-039|658[0n]bp|France.Ile-de-France|Mycetophagidae
Eulagius filicornis|BC-PNEF-PSFOR0323|360[0n]bp|France|Mycetophagidae
Eulagius filicornis|BC-PNEF-PSFOR0321|609[0n]bp|France|Mycetophagidae
Chrysanthia viridissima|BC-PNEF-PSFOR0529|658[0n]bp|France.Provence-Alpes-Cote d'Azur|Oedemeridae
Ischnomera caerulea|PASSIFOR-NM13-002|658[0n]bp|France.Ile-de-France|Oedemeridae
Ischnomera cyanea|BC-PNEF-PSFOR0532|381[0n]bp|France.Ile-de-France|Oedemeridae
Ischnomera cyanea|PASSIFOR-NM13-087|581[0n]bp|France.Ile-de-France|Oedemeridae
Hallomenus binotatus|BC-PASSIFOR0113|658[0n]bp|France.Rhone-Alpes|Tetratomidae
Cortodera femorata|BC-PNEF-PSFOR0092|658[0n]bp|France.Provence-Alpes-Cote d'Azur|Cerambycidae
Cortodera humeralis|BC-PNEF-PSFOR0418|658[0n]bp|France|Cerambycidae
Cortodera humeralis|BC-PNEF-PSFOR0093|658[0n]bp|France.Languedoc-Roussillon|Cerambycidae
Cortodera humeralis|BC-PASSIFOR0025|658[0n]bp|France|Cerambycidae
Lissodema lituratum|BC-PNEF-PSFOR0657|658[0n]bp|France.Languedoc-Roussillon|Salpingidae
Asemum striatum|BC-PNEF-PSFOR0012|658[0n]bp|France.Provence-Alpes-Cote d'Azur|Cerambycidae
Asemum striatum|BC-PNEF-PSFOR0013|658[0n]bp|France.Provence-Alpes-Cote d'Azur|Cerambycidae
Oxymirus cursor|BC-PNEF-PSFOR0043|658[0n]bp|France.Languedoc-Roussillon|Cerambycidae
Anisorus quercus|BC-PNEF-PSFOR0008|658[0n]bp|France.Provence-Alpes-Cote d'Azur|Cerambycidae
Stenocorus meridianus|PASSIFOR-NM13-080|658[0n]bp|France.Ile-de-France|Cerambycidae

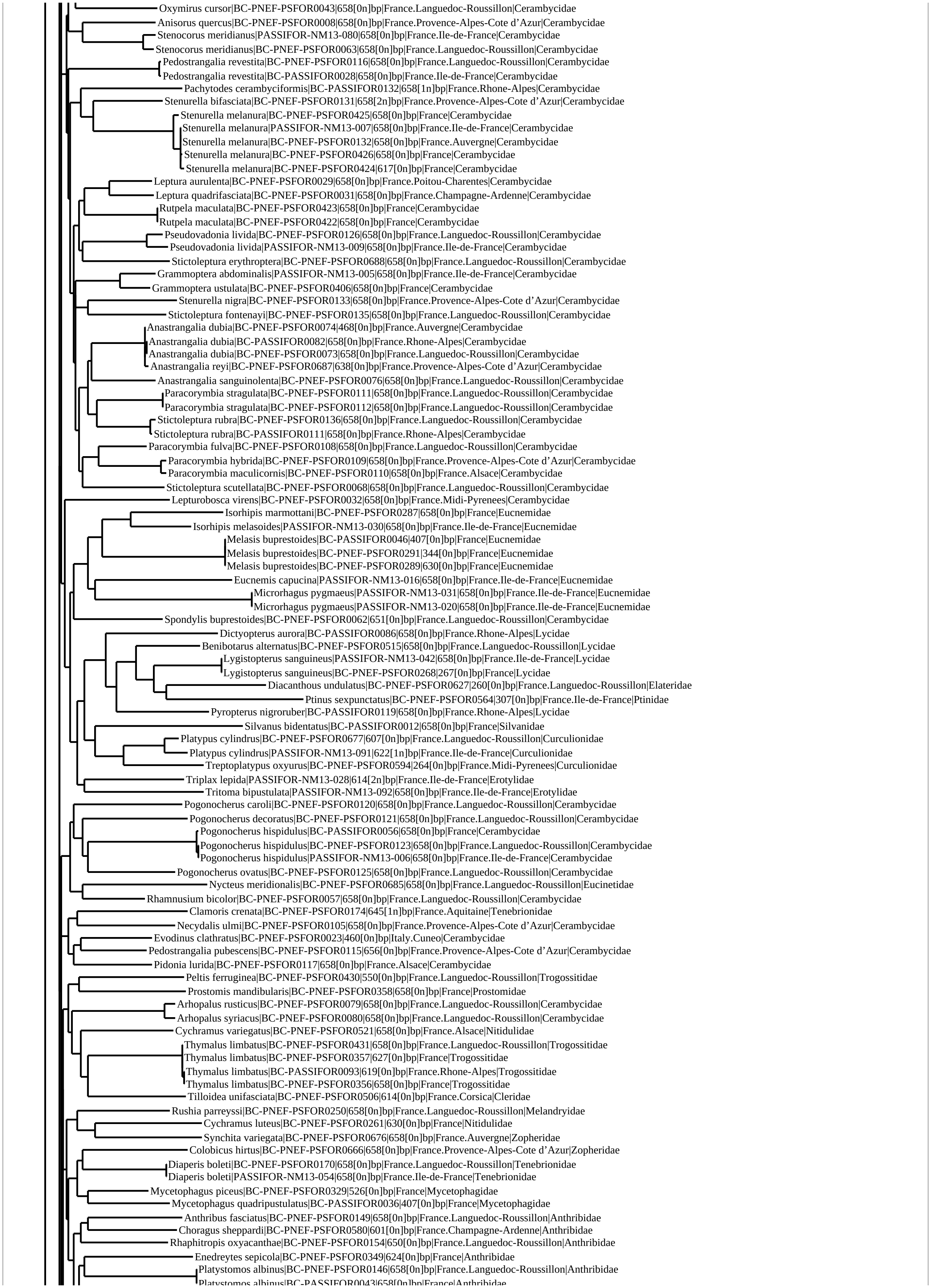
Oxymirus cursor|BC-PNEF-PSFOR0043|658[0n]bp|France.Languedoc-Roussillon|Cerambycidae
Anisorus quercus|BC-PNEF-PSFOR0008|658[0n]bp|France.Provence-Alpes-Cote d'Azur|Cerambycidae
Stenocorus meridianus|PASSIFOR-NM13-080|658[0n]bp|France.Ile-de-France|Cerambycidae
Stenocorus meridianus|BC-PNEF-PSFOR0063|658[0n]bp|France.Languedoc-Roussillon|Cerambycidae
Pedostrangalia revestita|BC-PNEF-PSFOR0116|658[0n]bp|France.Languedoc-Roussillon|Cerambycidae
Pedostrangalia revestita|BC-PASSIFOR0028|658[0n]bp|France.Ile-de-France|Cerambycidae
Pachytodes cerambyciformis|BC-PASSIFOR0132|658[1n]bp|France.Rhone-Alpes|Cerambycidae
Stenurella bifasciata|BC-PNEF-PSFOR0131|658[2n]bp|France.Provence-Alpes-Cote d'Azur|Cerambycidae
Stenurella melanura|BC-PNEF-PSFOR0425|658[0n]bp|France|Cerambycidae
Stenurella melanura|PASSIFOR-NM13-007|658[0n]bp|France.Ile-de-France|Cerambycidae
Stenurella melanura|BC-PNEF-PSFOR0132|658[0n]bp|France.Auvergne|Cerambycidae
Stenurella melanura|BC-PNEF-PSFOR0426|658[0n]bp|France|Cerambycidae
Stenurella melanura|BC-PNEF-PSFOR0424|617[0n]bp|France|Cerambycidae
Leptura aurulenta|BC-PNEF-PSFOR0029|658[0n]bp|France.Poitou-Charentes|Cerambycidae
Leptura quadrifasciata|BC-PNEF-PSFOR0031|658[0n]bp|France.Champagne-Ardenne|Cerambycidae
Rutpela maculata|BC-PNEF-PSFOR0423|658[0n]bp|France|Cerambycidae
Rutpela maculata|BC-PNEF-PSFOR0422|658[0n]bp|France|Cerambycidae
Pseudovadonia livida|BC-PNEF-PSFOR0126|658[0n]bp|France.Languedoc-Roussillon|Cerambycidae
Pseudovadonia livida|PASSIFOR-NM13-009|658[0n]bp|France.Ile-de-France|Cerambycidae
Stictoleptura erythroptera|BC-PNEF-PSFOR0688|658[0n]bp|France.Languedoc-Roussillon|Cerambycidae
Grammoptera abdominalis|PASSIFOR-NM13-005|658[0n]bp|France.Ile-de-France|Cerambycidae
Grammoptera ustulata|BC-PNEF-PSFOR0406|658[0n]bp|France|Cerambycidae
Stenurella nigra|BC-PNEF-PSFOR0133|658[0n]bp|France.Provence-Alpes-Cote d'Azur|Cerambycidae
Stictoleptura fontenayi|BC-PNEF-PSFOR0135|658[0n]bp|France.Languedoc-Roussillon|Cerambycidae
Anastrangalia dubia|BC-PNEF-PSFOR0074|468[0n]bp|France.Auvergne|Cerambycidae
Anastrangalia dubia|BC-PASSIFOR0082|658[0n]bp|France.Rhone-Alpes|Cerambycidae
Anastrangalia dubia|BC-PNEF-PSFOR0073|658[0n]bp|France.Languedoc-Roussillon|Cerambycidae
Anastrangalia reyi|BC-PNEF-PSFOR0687|638[0n]bp|France.Provence-Alpes-Cote d'Azur|Cerambycidae
Anastrangalia sanguinolenta|BC-PNEF-PSFOR0076|658[0n]bp|France.Languedoc-Roussillon|Cerambycidae
Paracorymbia stragulata|BC-PNEF-PSFOR0111|658[0n]bp|France.Languedoc-Roussillon|Cerambycidae
Paracorymbia stragulata|BC-PNEF-PSFOR0112|658[0n]bp|France.Languedoc-Roussillon|Cerambycidae
Stictoleptura rubra|BC-PNEF-PSFOR0136|658[0n]bp|France.Languedoc-Roussillon|Cerambycidae
Stictoleptura rubra|BC-PASSIFOR0111|658[0n]bp|France.Rhone-Alpes|Cerambycidae
Paracorymbia fulva|BC-PNEF-PSFOR0108|658[0n]bp|France.Languedoc-Roussillon|Cerambycidae
Paracorymbia hybrida|BC-PNEF-PSFOR0109|658[0n]bp|France.Provence-Alpes-Cote d'Azur|Cerambycidae
Paracorymbia maculicornis|BC-PNEF-PSFOR0110|658[0n]bp|France.Alsace|Cerambycidae
Stictoleptura scutellata|BC-PNEF-PSFOR0068|658[0n]bp|France.Languedoc-Roussillon|Cerambycidae
Lepturobosca virens|BC-PNEF-PSFOR0032|658[0n]bp|France.Midi-Pyrenees|Cerambycidae
Isorhipis marmottani|BC-PNEF-PSFOR0287|658[0n]bp|France|Eucnemidae
Isorhipis melasoides|PASSIFOR-NM13-030|658[0n]bp|France.Ile-de-France|Eucnemidae
Melasis buprestoides|BC-PASSIFOR0046|407[0n]bp|France|Eucnemidae
Melasis buprestoides|BC-PNEF-PSFOR0291|344[0n]bp|France|Eucnemidae
Melasis buprestoides|BC-PNEF-PSFOR0289|630[0n]bp|France|Eucnemidae
Eucnemis capucina|PASSIFOR-NM13-016|658[0n]bp|France.Ile-de-France|Eucnemidae
Microrhagus pygmaeus|PASSIFOR-NM13-031|658[0n]bp|France.Ile-de-France|Eucnemidae
Microrhagus pygmaeus|PASSIFOR-NM13-020|658[0n]bp|France.Ile-de-France|Eucnemidae
Spondylis buprestoides|BC-PNEF-PSFOR0062|651[0n]bp|France.Languedoc-Roussillon|Cerambycidae
Dictyopterus aurora|BC-PASSIFOR0086|658[0n]bp|France.Rhone-Alpes|Lycidae
Benibotarus alternatus|BC-PNEF-PSFOR0515|658[0n]bp|France.Languedoc-Roussillon|Lycidae
Lygistopterus sanguineus|PASSIFOR-NM13-042|658[0n]bp|France.Ile-de-France|Lycidae
Lygistopterus sanguineus|BC-PNEF-PSFOR0268|267[0n]bp|France|Lycidae
Diacanthous undulatus|BC-PNEF-PSFOR0627|260[0n]bp|France.Languedoc-Roussillon|Elateridae
Ptinus sexpunctatus|BC-PNEF-PSFOR0564|307[0n]bp|France.Ile-de-France|Ptinidae
Pyropterus nigroruber|BC-PASSIFOR0119|658[0n]bp|France.Rhone-Alpes|Lycidae
Silvanus bidentatus|BC-PASSIFOR0012|658[0n]bp|France|Silvanidae
Platypus cylindrus|BC-PNEF-PSFOR0677|607[0n]bp|France.Languedoc-Roussillon|Curculionidae
Platypus cylindrus|PASSIFOR-NM13-091|622[1n]bp|France.Ile-de-France|Curculionidae
Treptoplatypus oxyurus|BC-PNEF-PSFOR0594|264[0n]bp|France.Midi-Pyrenees|Curculionidae
Triplax lepida|PASSIFOR-NM13-028|614[2n]bp|France.Ile-de-France|Erotylidae
Tritoma bipustulata|PASSIFOR-NM13-092|658[0n]bp|France.Ile-de-France|Erotylidae
Pogonocherus caroli|BC-PNEF-PSFOR0120|658[0n]bp|France.Languedoc-Roussillon|Cerambycidae
Pogonocherus decoratus|BC-PNEF-PSFOR0121|658[0n]bp|France.Languedoc-Roussillon|Cerambycidae
Pogonocherus hispidulus|BC-PASSIFOR0056|658[0n]bp|France|Cerambycidae
Pogonocherus hispidulus|BC-PNEF-PSFOR0123|658[0n]bp|France.Languedoc-Roussillon|Cerambycidae
Pogonocherus hispidulus|PASSIFOR-NM13-006|658[0n]bp|France.Ile-de-France|Cerambycidae
Pogonocherus ovatus|BC-PNEF-PSFOR0125|658[0n]bp|France.Languedoc-Roussillon|Cerambycidae
Nycteus meridionalis|BC-PNEF-PSFOR0685|658[0n]bp|France.Languedoc-Roussillon|Eucinetidae
Rhamnusium bicolor|BC-PNEF-PSFOR0057|658[0n]bp|France.Languedoc-Roussillon|Cerambycidae
Clamoris crenata|BC-PNEF-PSFOR0174|645[1n]bp|France.Aquitaine|Tenebrionidae
Necydalis ulmi|BC-PNEF-PSFOR0105|658[0n]bp|France.Provence-Alpes-Cote d'Azur|Cerambycidae
Evodinus clathratus|BC-PNEF-PSFOR0023|460[0n]bp|Italy.Cuneo|Cerambycidae
Pedostrangalia pubescens|BC-PNEF-PSFOR0115|656[0n]bp|France.Provence-Alpes-Cote d'Azur|Cerambycidae
Pidonia lurida|BC-PNEF-PSFOR0117|658[0n]bp|France.Alsace|Cerambycidae
Peltis ferruginea|BC-PNEF-PSFOR0430|550[0n]bp|France.Languedoc-Roussillon|Trogossitidae
Prostomis mandibularis|BC-PNEF-PSFOR0358|658[0n]bp|France|Prostomidae
Arhopalus rusticus|BC-PNEF-PSFOR0079|658[0n]bp|France.Languedoc-Roussillon|Cerambycidae
Arhopalus syriacus|BC-PNEF-PSFOR0080|658[0n]bp|France.Languedoc-Roussillon|Cerambycidae
Cychramus variegatus|BC-PNEF-PSFOR0521|658[0n]bp|France.Alsace|Nitidulidae
Thymalus limbatus|BC-PNEF-PSFOR0431|658[0n]bp|France.Languedoc-Roussillon|Trogossitidae
Thymalus limbatus|BC-PNEF-PSFOR0357|627[0n]bp|France|Trogossitidae
Thymalus limbatus|BC-PASSIFOR0093|619[0n]bp|France.Rhone-Alpes|Trogossitidae
Thymalus limbatus|BC-PNEF-PSFOR0356|658[0n]bp|France|Trogossitidae
Tilloidea unifasciata|BC-PNEF-PSFOR0506|614[0n]bp|France.Corsica|Cleridae
Rushia parreyssi|BC-PNEF-PSFOR0250|658[0n]bp|France.Languedoc-Roussillon|Melandryidae
Cychramus luteus|BC-PNEF-PSFOR0261|630[0n]bp|France|Nitidulidae
Synchita variegata|BC-PNEF-PSFOR0676|658[0n]bp|France.Auvergne|Zopheridae
Colobicus hirtus|BC-PNEF-PSFOR0666|658[0n]bp|France.Provence-Alpes-Cote d'Azur|Zopheridae
Diaperis boleti|BC-PNEF-PSFOR0170|658[0n]bp|France.Languedoc-Roussillon|Tenebrionidae
Diaperis boleti|PASSIFOR-NM13-054|658[0n]bp|France.Ile-de-France|Tenebrionidae
Mycetophagus piceus|BC-PNEF-PSFOR0329|526[0n]bp|France|Mycetophagidae
Mycetophagus quadripustulatus|BC-PASSIFOR0036|407[0n]bp|France|Mycetophagidae
Anthribus fasciatus|BC-PNEF-PSFOR0149|658[0n]bp|France.Languedoc-Roussillon|Anthribidae
Choragus sheppardi|BC-PNEF-PSFOR0580|601[0n]bp|France.Champagne-Ardenne|Anthribidae
Rhaphitropis oxyacanthae|BC-PNEF-PSFOR0154|650[0n]bp|France.Languedoc-Roussillon|Anthribidae
Enedreytes sepicola|BC-PNEF-PSFOR0349|624[0n]bp|France|Anthribidae
Platystomos albinus|BC-PNEF-PSFOR0146|658[0n]bp|France.Languedoc-Roussillon|Anthribidae
Platystomos albinus|BC-PASSIFOR0043|658[0n]bp|France|Anthribidae

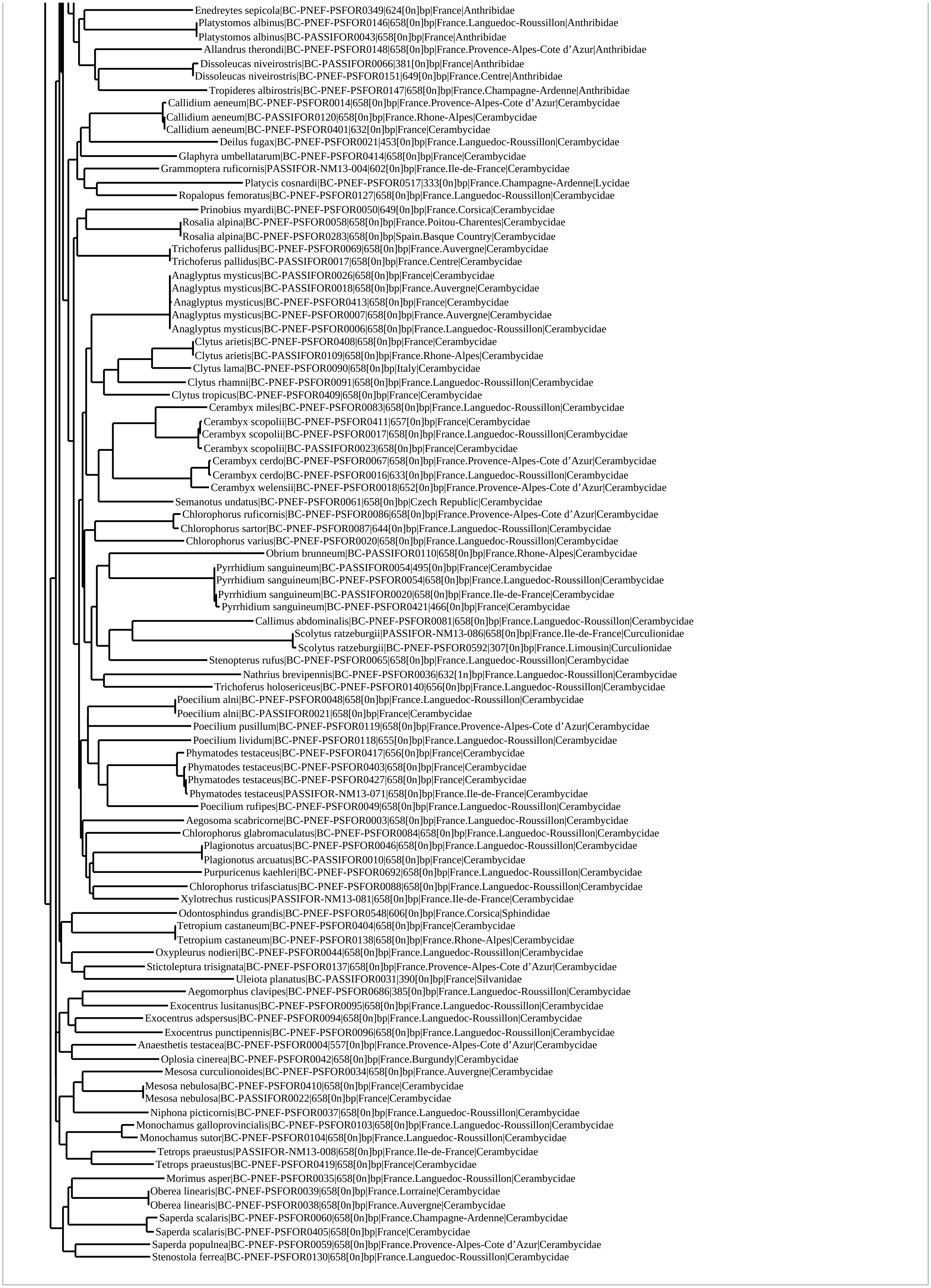
Enedreytes sepicola|BC-PNEF-PSFOR0349|624[0n]bp|France|Anthribidae
Platystomos albinus|BC-PNEF-PSFOR0146|658[0n]bp|France.Languedoc-Roussillon|Anthribidae
Platystomos albinus|BC-PASSIFOR0043|658[0n]bp|France|Anthribidae
Allandrus therondi|BC-PNEF-PSFOR0148|658[0n]bp|France.Provence-Alpes-Cote d'Azur|Anthribidae
Dissoleucas niveirostris|BC-PASSIFOR0066|381[0n]bp|France|Anthribidae
Dissoleucas niveirostris|BC-PNEF-PSFOR0151|649[0n]bp|France.Centre|Anthribidae
Tropideres albirostris|BC-PNEF-PSFOR0147|658[0n]bp|France.Champagne-Ardenne|Anthribidae
Callidium aeneum|BC-PNEF-PSFOR0014|658[0n]bp|France.Provence-Alpes-Cote d'Azur|Cerambycidae
Callidium aeneum|BC-PASSIFOR0120|658[0n]bp|France.Rhone-Alpes|Cerambycidae
Callidium aeneum|BC-PNEF-PSFOR0401|632[0n]bp|France|Cerambycidae
Deilus fugax|BC-PNEF-PSFOR0021|453[0n]bp|France.Languedoc-Roussillon|Cerambycidae
Glaphyra umbellatarum|BC-PNEF-PSFOR0414|658[0n]bp|France|Cerambycidae
Grammoptera ruficornis|PASSIFOR-NM13-004|602[0n]bp|France.Ile-de-France|Cerambycidae
Platycis cosnardi|BC-PNEF-PSFOR0517|333[0n]bp|France.Champagne-Ardenne|Lycidae
Ropalopus femoratus|BC-PNEF-PSFOR0127|658[0n]bp|France.Languedoc-Roussillon|Cerambycidae
Prinobius myardi|BC-PNEF-PSFOR0050|649[0n]bp|France.Corsica|Cerambycidae
Rosalia alpina|BC-PNEF-PSFOR0058|658[0n]bp|France.Poitou-Charentes|Cerambycidae
Rosalia alpina|BC-PNEF-PSFOR0283|658[0n]bp|Spain.Basque Country|Cerambycidae
Trichoferus pallidus|BC-PNEF-PSFOR0069|658[0n]bp|France.Auvergne|Cerambycidae
Trichoferus pallidus|BC-PASSIFOR0017|658[0n]bp|France.Centre|Cerambycidae
Anaglyptus mysticus|BC-PASSIFOR0026|658[0n]bp|France|Cerambycidae
Anaglyptus mysticus|BC-PASSIFOR0018|658[0n]bp|France.Auvergne|Cerambycidae
Anaglyptus mysticus|BC-PNEF-PSFOR0413|658[0n]bp|France|Cerambycidae
Anaglyptus mysticus|BC-PNEF-PSFOR0007|658[0n]bp|France.Auvergne|Cerambycidae
Anaglyptus mysticus|BC-PNEF-PSFOR0006|658[0n]bp|France.Languedoc-Roussillon|Cerambycidae
Clytus arietis|BC-PNEF-PSFOR0408|658[0n]bp|France|Cerambycidae
Clytus arietis|BC-PASSIFOR0109|658[0n]bp|France.Rhone-Alpes|Cerambycidae
Clytus lama|BC-PNEF-PSFOR0090|658[0n]bp|Italy|Cerambycidae
Clytus rhamni|BC-PNEF-PSFOR0091|658[0n]bp|France.Languedoc-Roussillon|Cerambycidae
Clytus tropicus|BC-PNEF-PSFOR0409|658[0n]bp|France|Cerambycidae
Cerambyx miles|BC-PNEF-PSFOR0083|658[0n]bp|France.Languedoc-Roussillon|Cerambycidae
Cerambyx scopolii|BC-PNEF-PSFOR0411|657[0n]bp|France|Cerambycidae
Cerambyx scopolii|BC-PNEF-PSFOR0017|658[0n]bp|France.Languedoc-Roussillon|Cerambycidae
Cerambyx scopolii|BC-PASSIFOR0023|658[0n]bp|France|Cerambycidae
Cerambyx cerdo|BC-PNEF-PSFOR0067|658[0n]bp|France.Provence-Alpes-Cote d'Azur|Cerambycidae
Cerambyx cerdo|BC-PNEF-PSFOR0016|633[0n]bp|France.Languedoc-Roussillon|Cerambycidae
Cerambyx welensii|BC-PNEF-PSFOR0018|652[0n]bp|France.Provence-Alpes-Cote d'Azur|Cerambycidae
Semanotus undatus|BC-PNEF-PSFOR0061|658[0n]bp|Czech Republic|Cerambycidae
Chlorophorus ruficornis|BC-PNEF-PSFOR0086|658[0n]bp|France.Provence-Alpes-Cote d'Azur|Cerambycidae
Chlorophorus sartor|BC-PNEF-PSFOR0087|644[0n]bp|France.Languedoc-Roussillon|Cerambycidae
Chlorophorus varius|BC-PNEF-PSFOR0020|658[0n]bp|France.Languedoc-Roussillon|Cerambycidae
Obrium brunneum|BC-PASSIFOR0110|658[0n]bp|France.Rhone-Alpes|Cerambycidae
Pyrrhidium sanguineum|BC-PASSIFOR0054|495[0n]bp|France|Cerambycidae
Pyrrhidium sanguineum|BC-PNEF-PSFOR0054|658[0n]bp|France.Languedoc-Roussillon|Cerambycidae
Pyrrhidium sanguineum|BC-PASSIFOR0020|658[0n]bp|France.Ile-de-France|Cerambycidae
Pyrrhidium sanguineum|BC-PNEF-PSFOR0421|466[0n]bp|France|Cerambycidae
Callimus abdominalis|BC-PNEF-PSFOR0081|658[0n]bp|France.Languedoc-Roussillon|Cerambycidae
Scolytus ratzeburgii|PASSIFOR-NM13-086|658[0n]bp|France.Ile-de-France|Curculionidae
Scolytus ratzeburgii|BC-PNEF-PSFOR0592|307[0n]bp|France.Limousin|Curculionidae
Stenopterus rufus|BC-PNEF-PSFOR0065|658[0n]bp|France.Languedoc-Roussillon|Cerambycidae
Nathrius brevipennis|BC-PNEF-PSFOR0036|632[1n]bp|France.Languedoc-Roussillon|Cerambycidae
Trichoferus holosericeus|BC-PNEF-PSFOR0140|656[0n]bp|France.Languedoc-Roussillon|Cerambycidae
Poecilium alni|BC-PNEF-PSFOR0048|658[0n]bp|France.Languedoc-Roussillon|Cerambycidae
Poecilium alni|BC-PASSIFOR0021|658[0n]bp|France|Cerambycidae
Poecilium pusillum|BC-PNEF-PSFOR0119|658[0n]bp|France.Provence-Alpes-Cote d'Azur|Cerambycidae
Poecilium lividum|BC-PNEF-PSFOR0118|655[0n]bp|France.Languedoc-Roussillon|Cerambycidae
Phymatodes testaceus|BC-PNEF-PSFOR0417|656[0n]bp|France|Cerambycidae
Phymatodes testaceus|BC-PNEF-PSFOR0403|658[0n]bp|France|Cerambycidae
Phymatodes testaceus|BC-PNEF-PSFOR0427|658[0n]bp|France|Cerambycidae
Phymatodes testaceus|PASSIFOR-NM13-071|658[0n]bp|France.Ile-de-France|Cerambycidae
Poecilium rufipes|BC-PNEF-PSFOR0049|658[0n]bp|France.Languedoc-Roussillon|Cerambycidae
Aegosoma scabricorne|BC-PNEF-PSFOR0003|658[0n]bp|France.Languedoc-Roussillon|Cerambycidae
Chlorophorus glabromaculatus|BC-PNEF-PSFOR0084|658[0n]bp|France.Languedoc-Roussillon|Cerambycidae
Plagionotus arcuatus|BC-PNEF-PSFOR0046|658[0n]bp|France.Languedoc-Roussillon|Cerambycidae
Plagionotus arcuatus|BC-PASSIFOR0010|658[0n]bp|France|Cerambycidae
Purpuricenus kaehleri|BC-PNEF-PSFOR0692|658[0n]bp|France.Languedoc-Roussillon|Cerambycidae
Chlorophorus trifasciatus|BC-PNEF-PSFOR0088|658[0n]bp|France.Languedoc-Roussillon|Cerambycidae
Xylotrechus rusticus|PASSIFOR-NM13-081|658[0n]bp|France.Ile-de-France|Cerambycidae
Odontosphindus grandis|BC-PNEF-PSFOR0548|606[0n]bp|France.Corsica|Sphindidae
Tetropium castaneum|BC-PNEF-PSFOR0404|658[0n]bp|France|Cerambycidae
Tetropium castaneum|BC-PNEF-PSFOR0138|658[0n]bp|France.Rhone-Alpes|Cerambycidae
Oxypleurus nodieri|BC-PNEF-PSFOR0044|658[0n]bp|France.Languedoc-Roussillon|Cerambycidae
Stictoleptura trisignata|BC-PNEF-PSFOR0137|658[0n]bp|France.Provence-Alpes-Cote d'Azur|Cerambycidae
Uleiota planatus|BC-PASSIFOR0031|390[0n]bp|France|Silvanidae
Aegomorphus clavipes|BC-PNEF-PSFOR0686|385[0n]bp|France.Languedoc-Roussillon|Cerambycidae
Exocentrus lusitanus|BC-PNEF-PSFOR0095|658[0n]bp|France.Languedoc-Roussillon|Cerambycidae
Exocentrus adspersus|BC-PNEF-PSFOR0094|658[0n]bp|France.Languedoc-Roussillon|Cerambycidae
Exocentrus punctipennis|BC-PNEF-PSFOR0096|658[0n]bp|France.Languedoc-Roussillon|Cerambycidae
Anaesthetis testacea|BC-PNEF-PSFOR0004|557[0n]bp|France.Provence-Alpes-Cote d'Azur|Cerambycidae
Oplosia cinerea|BC-PNEF-PSFOR0042|658[0n]bp|France.Burgundy|Cerambycidae
Mesosa curculionoides|BC-PNEF-PSFOR0034|658[0n]bp|France.Auvergne|Cerambycidae
Mesosa nebulosa|BC-PNEF-PSFOR0410|658[0n]bp|France|Cerambycidae
Mesosa nebulosa|BC-PASSIFOR0022|658[0n]bp|France|Cerambycidae
Niphona picticornis|BC-PNEF-PSFOR0037|658[0n]bp|France.Languedoc-Roussillon|Cerambycidae
Monochamus galloprovincialis|BC-PNEF-PSFOR0103|658[0n]bp|France.Languedoc-Roussillon|Cerambycidae
Monochamus sutor|BC-PNEF-PSFOR0104|658[0n]bp|France.Languedoc-Roussillon|Cerambycidae
Tetrops praeustus|PASSIFOR-NM13-008|658[0n]bp|France.Ile-de-France|Cerambycidae
Tetrops praeustus|BC-PNEF-PSFOR0419|658[0n]bp|France|Cerambycidae
Morimus asper|BC-PNEF-PSFOR0035|658[0n]bp|France.Languedoc-Roussillon|Cerambycidae
Oberea linearis|BC-PNEF-PSFOR0039|658[0n]bp|France.Lorraine|Cerambycidae
Oberea linearis|BC-PNEF-PSFOR0038|658[0n]bp|France.Auvergne|Cerambycidae
Saperda scalaris|BC-PNEF-PSFOR0060|658[0n]bp|France.Champagne-Ardenne|Cerambycidae
Saperda scalaris|BC-PNEF-PSFOR0405|658[0n]bp|France|Cerambycidae
Saperda populnea|BC-PNEF-PSFOR0059|658[0n]bp|France.Provence-Alpes-Cote d'Azur|Cerambycidae
Stenostola ferrea|BC-PNEF-PSFOR0130|658[0n]bp|France.Languedoc-Roussillon|Cerambycidae
